# A maladaptive feedback mechanism between the extracellular matrix and cytoskeleton contributes to hypertrophic cardiomyopathy pathophysiology

**DOI:** 10.1038/s42003-022-04278-9

**Published:** 2023-01-03

**Authors:** Helena M. Viola, Caitlyn Richworth, Tanya Solomon, Ian L. Chin, Henrietta Cserne Szappanos, Srinivasan Sundararaj, Dmitry Shishmarev, Marco G. Casarotto, Yu Suk Choi, Livia C. Hool

**Affiliations:** 1 School of Human Sciences, The University of Western Australia, Crawley, WA Australia; 2 The Australian National University, Canberra, ACT Australia; 3 Victor Chang Cardiac Research Institute, Sydney, NSW Australia

**Keywords:** Preclinical research, Cardiac hypertrophy

## Abstract

Hypertrophic cardiomyopathy is an inherited disorder due to mutations in contractile proteins that results in a stiff, hypercontractile myocardium. To understand the role of cardiac stiffness in disease progression, here we create an in vitro model of hypertrophic cardiomyopathy utilizing hydrogel technology. Culturing wild-type cardiac myocytes on hydrogels with a Young’s Moduli (stiffness) mimicking hypertrophic cardiomyopathy myocardium is sufficient to induce a hypermetabolic mitochondrial state versus myocytes plated on hydrogels simulating healthy myocardium. Significantly, these data mirror that of myocytes isolated from a murine model of human hypertrophic cardiomyopathy (*cTnI-G203S*). Conversely, *cTnI-G203S* myocyte mitochondrial function is completely restored when plated on hydrogels mimicking healthy myocardium. We identify a mechanosensing feedback mechanism between the extracellular matrix and cytoskeletal network that regulates mitochondrial function under healthy conditions, but participates in the progression of hypertrophic cardiomyopathy pathophysiology resulting from sarcomeric gene mutations. Importantly, we pinpoint key ‘linker’ sites in this schema that may represent potential therapeutic targets.

## Abstract (web summary)

An in vitro model of hypertrophic cardiomyopathy is developed and used to identify a structural-functional link between the L-type calcium channel, mitochondria and the extracellular matrix.

## Introduction

Hypertrophic cardiomyopathy (HCM) is an autosomal dominant genetic heart disease that affects 1:500 of the general population^1^. It is the leading cause of sudden cardiac death in the young (5–15 year olds)^2^. HCM occurs predominantly due to genetic mutations in sarcomeric proteins^3,4^. Clinical characteristics of HCM include left ventricular hypertrophy in the absence of increased hemodynamic workload, and a non-dilated left ventricle with preserved or increased ejection fraction^1,4^. At the cellular level, HCM is characterized by cardiac myocyte remodeling, sarcomeric protein disorganization, interstitial fibrosis, and altered energy metabolism^5^. We have previously identified a role for the cardiac L-type calcium channel (LTCC) in the development of HCM pathophysiology^6,7^.

The cardiac LTCC triggers “calcium-induced calcium release” that is critical to maintaining cardiac excitation–contraction coupling^8^. The cardiac LTCC also plays an important role in regulating mitochondrial function via both calcium-dependent and calcium-independent mechanisms^9,10^. Specifically, activation of the LTCC evokes an increase in mitochondrial membrane potential (*Ψ*_m_), in the presence or absence of calcium^9^. This is facilitated by a structural–functional association between the channel and mitochondria. The cardiac LTCC is a transmembrane spanning protein comprised of α_1C_, α_2_δ, and β_2_ subunits. The β_2_ subunit is bound to the pore-forming α_1C_ subunit via the α-interaction domain (AID)^11^. The β_2_ subunit also associates with neuroblast differentiation-associated protein AHNAK, also known as desmoyokin, a large subsarcolemmal protein, that is also anchored to F-actin^12^. Cytoskeletal proteins, including F-actin, interact directly with mitochondria by binding to outer mitochondrial docking proteins^13–15^. This structural link between the LTCC and mitochondria plays an important role in regulating mitochondrial function under physiological conditions, with conformational changes that occur in the LTCC β_2_ subunit on a beat-to-beat basis leading to downstream alterations in mitochondrial function^9^.

Impairments to this intracellular network are associated with dysregulation of mitochondrial function and the development of pathological states^6,7,16,17^. We have previously shown that a murine model of human HCM causing troponin I (cTnI) gene mutation Gly203Ser (*cTnI-G203S*) exhibits a disrupted cytoskeletal architecture, *impaired* structural–functional communication between the LTCC and mitochondria, and a resulting hypermetabolic mitochondrial state^6,17^. Similar findings have been recorded in mice carrying the human disease causing β-myosin heavy chain mutation Arg403Gln^7^. Notably, these responses preceded development of the hypertrophic state.

The established human HCM phenotype is characterized by a stiff myocardium^18,19^. In line with this, increased extracellular matrix (ECM) protein synthesis has been implicated in the progression of human HCM pathology^20–22^. Indeed, engineered heart tissues created by seeding healthy human-induced pluripotent stem cell-derived cardiac myocytes onto strips of decellularized myocardium harvested from a swine model of HCM exhibit increased stiffness^21^. The ECM interacts with cardiac myocytes via transmembrane spanning mechanosensing protein integrin, that has also been implicated in the development of cardiac hypertrophy^23,24^. Cytosolic protein vinculin mediates binding of integrin to F-actin, and transfers mechanical forces from the cell membrane to the cytoskeleton^23,25,26^. Therefore, in addition to an intracellular network between the LTCC and mitochondria, a structural–functional association also appears to exist between the ECM and F-actin. Here we explore the role of this link in the development of HCM pathophysiology involving the LTCC and mitochondria by creating an in vitro platform that mimics HCM myocardial stiffness.

## Results

### An in vitro model of cardiac myocyte stiffness

**Fig. 1:**
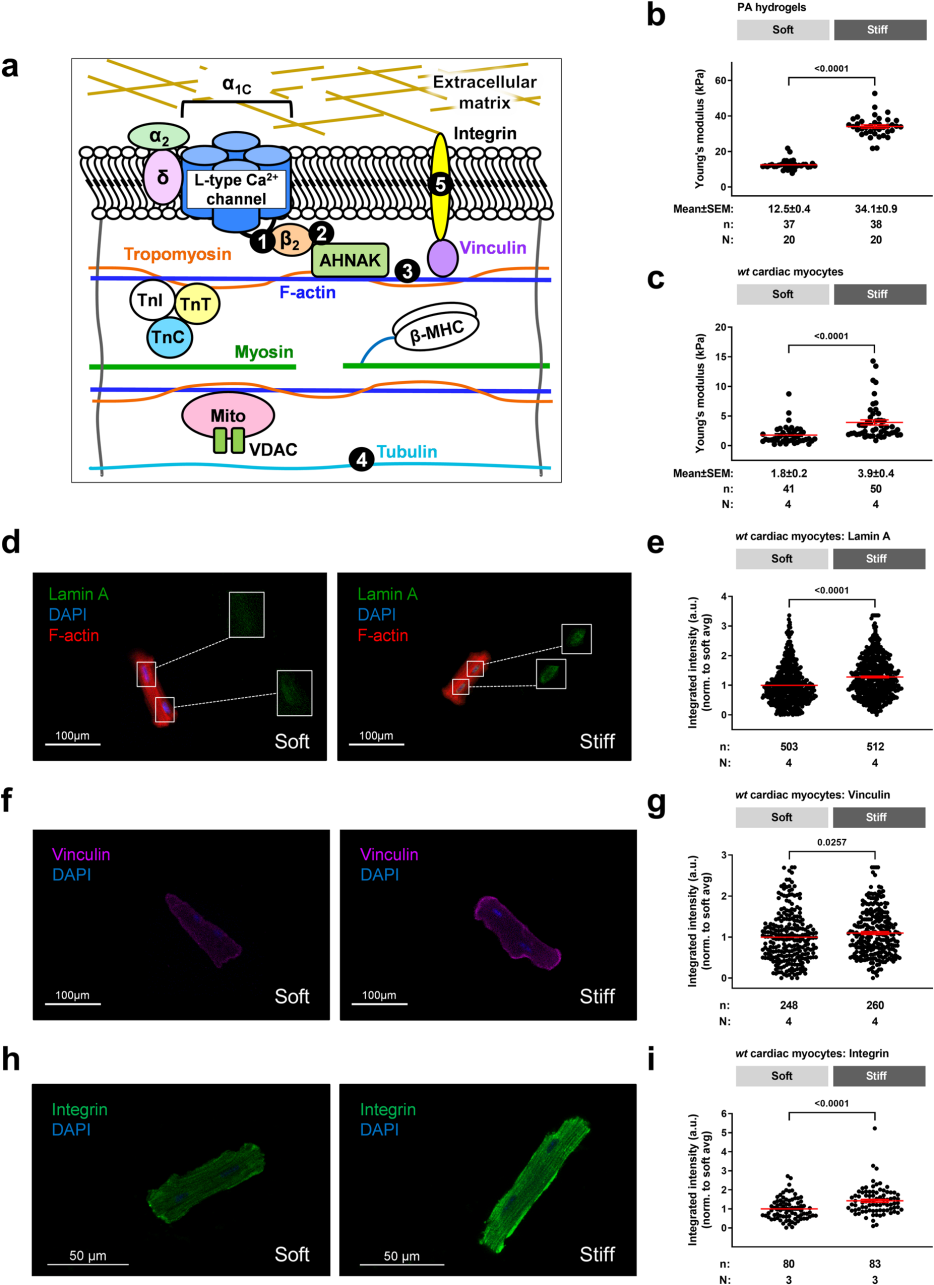
An in vitro platform that mimics HCM myocardial stiffness. **a** Schematic indicating structural-functional link between the L-type calcium channel, cytoskeletal network and mitochondria in *wt* cardiac myocytes. Targeted linker sites (1) L-type calcium channel α-interaction domain (AID), (2) interaction site between the L-type calcium channel β_2_ subunit and AHNAK, (3) F-actin, (4) β-tubulin, and (5) β_1_ integrin. **b** Compressive stiffness (in kPa) of polyacrylamide (PA) hydrogels under manufactured conditions (soft and stiff), including mean ± SEM. **c** Compressive stiffness (in kPa) of *wt* cardiac myocytes plated on soft and stiff hydrogels, including mean ± SEM. Representative images and quantitated data points for *wt* cardiac myocytes plated on soft and stiff hydrogels stained for lamin A (**d**, **e**), vinculin (**f**, **g**), and integrin (**h**, **i**), including mean ± SEM. *n* = number of cells, *N* = number of hydrogels. Statistical significance determined by Mann Whitney tests.

We sought to develop an in vitro model of hypertrophic cardiomyopathy. We began by assessing the compressive Young’s Moduli (stiffness) of hydrogels produced to mimic healthy (“soft”) and HCM (“stiff”) myocardium. Using atomic force microscopy, the overall compressive stiffness of hydrogels was recorded at 12.5 ± 0.4 and 34.1 ± 0.9 kPa respectively (*p* < 0.05, Fig. 1b). We then utilized a H9C2 myoblast cell line isolated from rat ventricular tissue, to test our in vitro platform. H9C2 cells were plated on hydrogel substrates coated with ECM protein laminin. H9C2 cells plated on stiff hydrogels displayed a significant increase in compressive stiffness versus soft hydrogels (*p* < 0.05, Supplementary Fig. 1a). Additionally, immunohistochemistry studies revealed a significant increase in lamin A, a cellular marker of stiffness, as well as mechanosensing protein vinculin (*p* < 0.05, Supplementary Fig. 1b–e)^27–29^. These data indicate that altering the compressive stiffness of the hydrogel platform was sufficient to alter the stiffness of the cells.

Next, we performed studies on cardiac myocytes isolated from 10 to 15-week-old wild-type (*wt*) mice. Myocytes plated on laminin coated soft and stiff hydrogel substrates yielded stiffness’s of approximately 2 and 4 kPa respectively (*p* < 0.05, Fig. 1c). Importantly, these values align with previously reported measurements of myocardial stiffness assessed in 20–40 year old healthy and HCM adults^18^. Myocytes plated on stiff hydrogels also displayed a significant increase in lamin A, vinculin and β_1_-integrin, the most abundantly expressed integrin isoform in the post-natal heart^23,30^ (*p* < 0.05, Fig. 1d–i). These data indicate that our in vitro platform is suitably representative of the human condition.

### wt cardiac myocytes plated on stiff hydrogels exhibit a hypermetabolic mitochondrial state

**Fig. 2:**
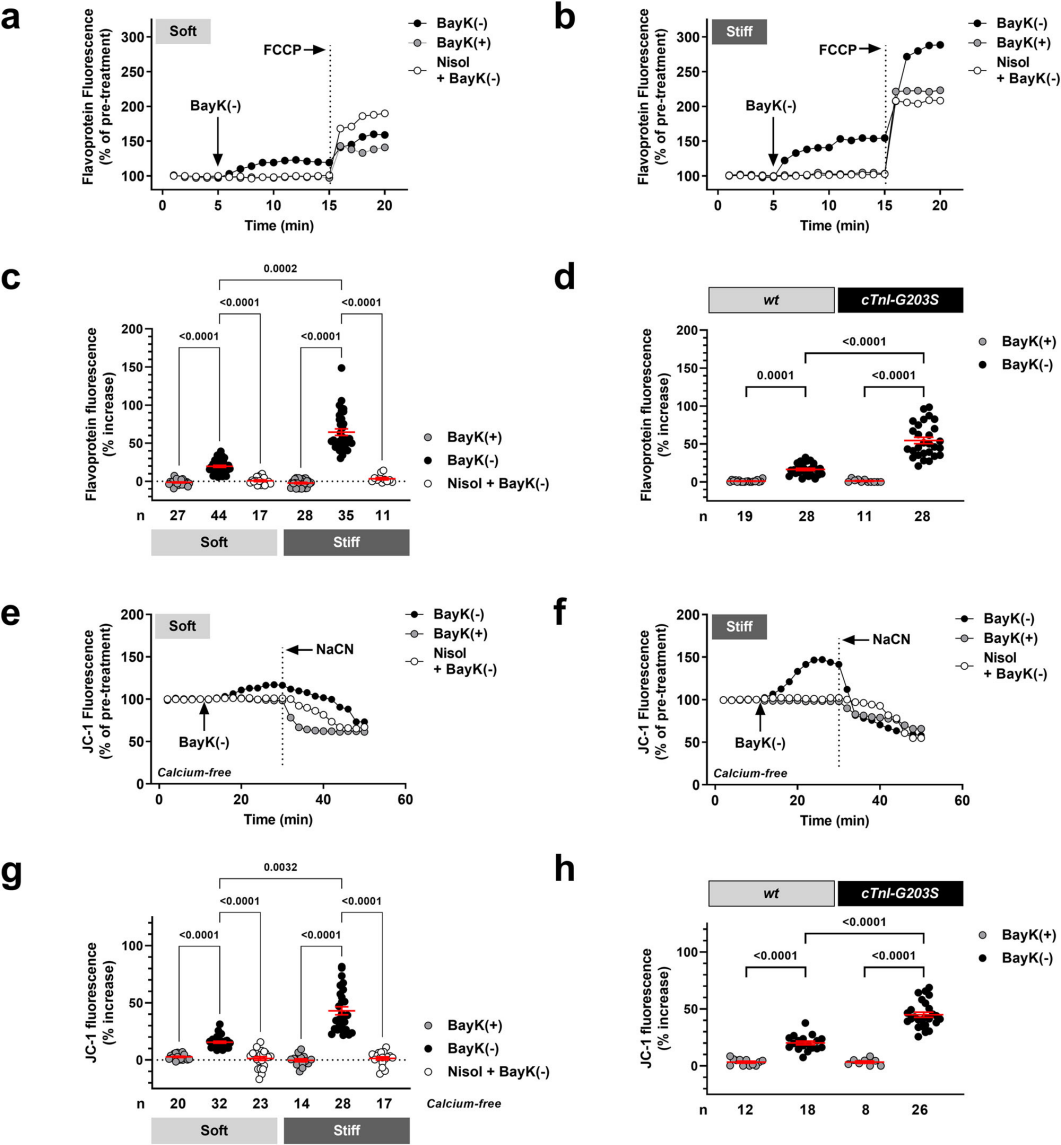
Increased ECM stiffness contributes to the progression of HCM pathophysiology. Representative ratiometric flavoprotein (**a**, **b**) and JC-1 (**e**, **f**) fluorescence recorded from *wt* cardiac myocytes plated on soft and stiff hydrogels before and after exposure to 10 μM BayK(+), or 10 μM BayK(−) in the absence or presence of nisoldipine (15 μM, Nisol). JC-1 studies were performed under calcium-free conditions (0 mM calcium). Arrows indicate addition of drugs. To confirm signals were mitochondrial in origin, FCCP (50 μM) was applied at the end of each flavoprotein experiment to increase flavoprotein oxidation. NaCN (40 mM) was applied at the end of each JC-1 experiment to collapse Ψ_m_. Overall flavoprotein (**c**) and JC-1 (**d**) fluorescence for all myocytes (*n*) exposed to drugs as indicated, including mean ± SEM. **d**–**h**, flavoprotein (**d**) and JC-1 (**h**) fluorescence for cardiac myocytes (*n*, in suspension) isolated from cardiomyopathic *cTnI-G203S* mice and *wt* counterparts after exposure to BayK(+) or 10 μM BayK(−)^17^. All statistical significance determined by Kruskal-Wallis tests, or Browne-Forsythe and Welch ANOVA test (**i**).

We have previously demonstrated that *cTnI-G203S* cardiac myocytes exhibit increased mitochondrial metabolic activity and *Ψ*_m_ that is consistent with the human condition^31^, in response to activation of the LTCC^6,17^. Here, we explored if simply plating *wt* cardiac myocytes on hydrogels with a stiffness mimicking HCM myocardium was sufficient to mirror these responses. We tested the effect of substrate stiffness on mitochondrial metabolic activity and *Ψ*_m_ in cardiac myocytes isolated from 10 to 15-week-old *wt* mice, in response to activation of the LTCC using channel agonist BayK(−). Metabolic activity is dependent upon oxygen consumption and mitochondrial electron transport down the electron transport chain. Therefore, we measured alterations in mitochondrial electron transport in intact cardiac myocytes plated on soft and stiff hydrogels for at least 3 h, by monitoring changes in flavoprotein oxidation (as autofluorescence). We found that *wt* myocytes plated on soft and stiff hydrogels both showed a significant increase in flavoprotein oxidation following exposure to BayK(−) versus the (+)enantiomer of BayK that does not act as an agonist (BayK(+)) (Fig. 2a–c). However the magnitude of the BayK(−) response was significantly larger on stiff versus soft hydrogels. Importantly, all BayK(−) induced responses could be attenuated with application of LTCC antagonist nisoldipine, confirming that the LTCC mediated the response. These data indicate that alterations in ECM stiffness are sufficient to alter regulation of mitochondrial function by the LTCC.

In order to examine the effect of substrate stiffness on the structural-functional communication between the channel and mitochondria, we also assessed alterations in *Ψ*_m_ under calcium-free conditions. Although oxidative phosphorylation is a calcium-dependent process, *Ψ*_m_ remains highly polarized under conditions of low intracellular calcium (0–535 nM)^32,33^. Indeed, studies performed in mouse and guinea pig cardiac myocytes demonstrate that activation of the LTCC by voltage-clamp of the plasma membrane, application of high K^+^ solution or BayK(−), results in an increase in *Ψ*_m_, even in the absence of calcium^9^. Depolymerization of actin or β-tubulin with Latrunculin A or colchicine, or the absence of dystrophin, attenuates elevated *Ψ*_m_ indicating that the response is dependent on an intact cytoskeletal architecture^6,7,9,16^. Here, consistent with observed alterations in flavoprotein oxidation, we found that *wt* myocytes plated on soft and stiff hydrogels, incubated for at least 3 h in calcium-free and EGTA containing HBS, both displayed a significant increase in *Ψ*_m_ following application of BayK(−) versus BayK(+) (Fig. 2e–g). Once again, the magnitude of the BayK(−) response was significantly larger on stiff versus soft hydrogels. All BayK(–) induced responses were abolished with nisoldipine, confirming that the LTCC mediated the response. These data indicate that alterations in ECM stiffness are sufficient to alter the structural-functional communication between the LTCC and mitochondria.

Significantly, the alterations in metabolic activity and *Ψ*_m_ observed in *wt* myocytes plated on stiff versus soft hydrogels mimicked previous data obtained from *cTnI-G203S* versus *wt* cardiac myocytes (Fig. 2d, h)^6,17^. These data indicate that increased ECM stiffness may contribute to the progression of HCM pathophysiology.

### Unraveling the structural–functional link between the LTCC, mitochondria, and ECM

**Fig. 3:**
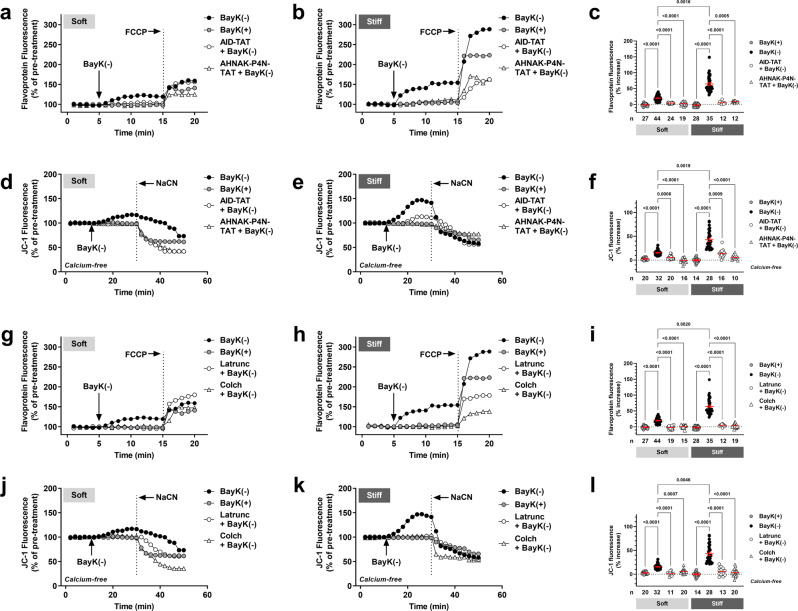
A key structural–functional network between the cardiac L-type calcium channel and mitochondria assists in regulating mitochondrial function under conditions of varying substrate stiffness. Representative ratiometric flavoprotein (**a**, **b** and **g**, **h**) and JC-1 (**d**, **e** and **j**, **k**) fluorescence recorded from *wt* cardiac myocytes plated on soft and stiff hydrogels before and after exposure to 10 μM BayK(+), or 10 μM BayK(−) in the absence or presence of AID-TAT (10 μM), AHNAK-P4N-TAT (1 μM), Latrunculin A (Latrunc, 5 μM, 20 min pre-incubation) or Colchicine (Colch, 1 μM, 3 h pre-incubation^51^). JC-1 studies were performed under calcium-free conditions (0 mM calcium). Arrows indicate addition of drugs. To confirm signals were mitochondrial in origin, FCCP (50 μM) was applied at the end of each flavoprotein experiment to increase flavoprotein oxidation. NaCN (40 mM) was applied at the end of each JC-1 experiment to collapse *Ψ*_m_. Overall flavoprotein (**c** and **i**) and JC-1 (**f** and **l**) fluorescence for all myocytes (*n*) exposed to drugs as indicated, including mean ± SEM. All statistical significance determined by Kruskal-Wallis tests.

We examined various “linker” sites between the LTCC and mitochondria as potential mediators of mitochondrial function in response to increased substrate stiffness. We found that BayK(−) induced increases in both flavoprotein oxidation and Ψ_m_ were significantly attenuated in *wt* myocytes plated on soft and stiff hydrogels in the presence of a peptide derived specifically against the cardiac LTCC AID that immobilizes the LTCC β_2_ subunit (AID-TAT peptide, Fig. 1a) (Fig. 3a–f). Similar results were observed in the presence of a peptide that targets the interaction site between the LTCC β_2_ subunit and AHNAK (AHNAK-P4N-TAT, Fig. 1a) (Fig. 3a–f). Similarly, BayK(−) induced alterations in both flavoprotein oxidation and Ψ_m_ were abolished in the presence of actin or β-tubulin depolymerizing agents Latrunculin A or colchicine respectively (Fig. 3g–l). These data confirm a key structural-functional network between the cardiac LTCC and mitochondria *via* the cytoskeletal network that regulates mitochondrial function under conditions of altered ECM stiffness.

**Fig. 4:**
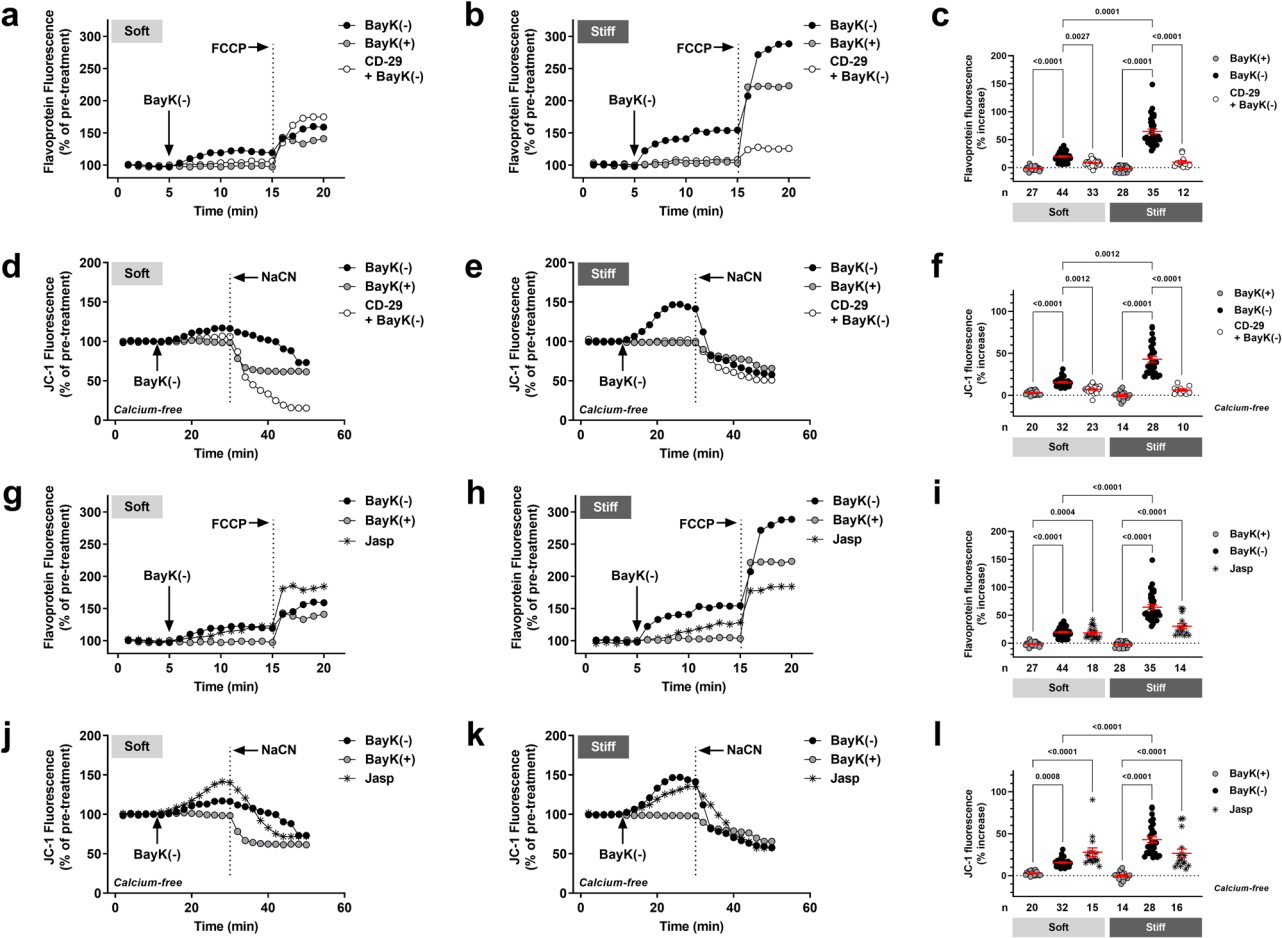
The cardiac LTCC and integrin participate as partners in mediating mitochondrial function under conditions of varying substrate stiffness. Representative ratiometric flavoprotein (**a**, **b** and **g**, **h**) and JC-1 (**d**, **e** and **j**, **k**) fluorescence recorded from *wt* cardiac myocytes plated on soft and stiff hydrogels before and after exposure to 10 μM BayK(+), Jasplakinolide (Jasp, 1 μM, 20 min pre-incubation), or 10 μM BayK(−) in the absence or presence of CD-29 (5 μg/ml, 30 min pre-incubation). JC-1 studies were performed under calcium-free conditions (0 mM calcium). Arrows indicate addition of drugs. To confirm signals were mitochondrial in origin, FCCP (50 μM) was applied at the end of each flavoprotein experiment to increase flavoprotein oxidation. NaCN (40 mM) was applied at the end of each JC-1 experiment to collapse *Ψ*_m_. Overall flavoprotein (**c** and **i**) and JC-1 (**f** and **l**) fluorescence for all myocytes (*n*) exposed to drugs as indicated, including mean ± SEM. All statistical significance determined by Kruskal-Wallis tests.

In addition to an intracellular link between the LTCC and mitochondria via the cytoskeletal network, a structural-functional association also appears to exist between the ECM and F-actin via integrin. Interestingly, here we find that BayK(−) induced alterations in both flavoprotein oxidation and *Ψ*_m_ were significantly attenuated in *wt* myocytes plated on soft and stiff hydrogels in the presence of a murine β_1_-integrin function blocking antibody (CD-29, Fig. 1a) (Fig. 4a–f). Overall, these data suggest that the cardiac LTCC and integrin may participate as partners in mediating mitochondrial function under conditions of ECM stiffness mimicking the healthy and HCM phenotype. We further explored this network by manipulating intracellular stiffness. We found that exposure of *wt* myocytes plated on soft and stiff hydrogels to jasplakinolide, a drug that stabilizes F-actin and thereby stiffens the cytoskeletal network^34^, was sufficient to induce a significant increase in flavoprotein oxidation and *Ψ*_m_ (Fig. 4g–l), indicating a potential role of cytoskeletal stiffness in regulating mitochondrial function under both conditions. These findings are supported by studies demonstrating that alterations in cytoskeletal proteins are sufficient to influence mitochondrial function^13–15^.

### Decreasing substrate stiffness is sufficient to reverse the cTnI-G203S hypermetabolic mitochondrial state

**Fig. 5:**
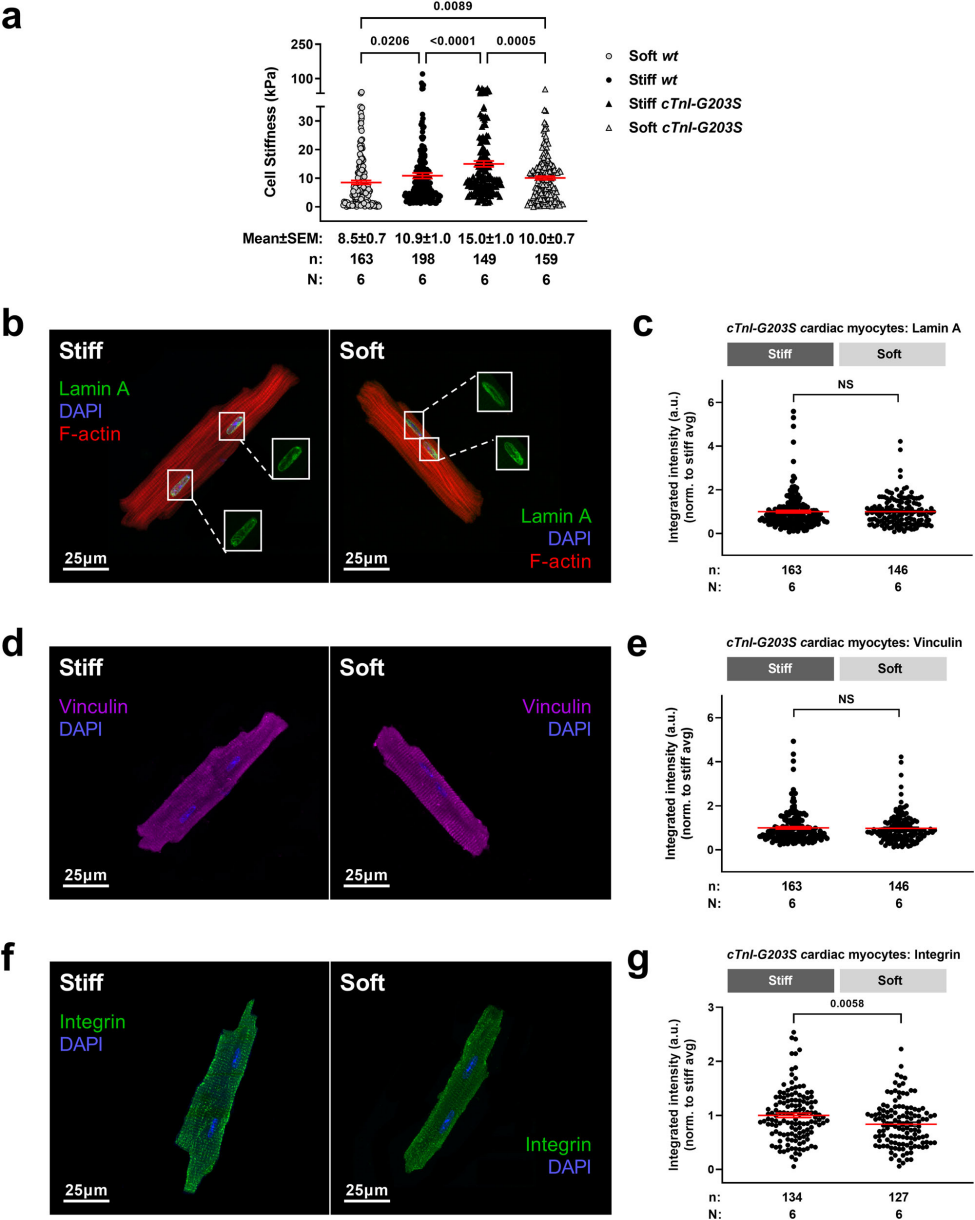
Integrin is a key mechanosensing protein in cTnI-G203S myocytes. **a** Compressive stiffness (in kPa) of cardiac myocytes isolated from cardiomyopathic *cTnI-G203S* mice and *wt* counterparts plated on soft and stiff hydrogels, including mean ± SEM. **b**–**g** Representative images and quantitated data points for *cTnI-G203S* cardiac myocytes plated on stiff and soft hydrogels stained for lamin A (**b**, **c**), vinculin (**d**, **e**), and integrin (**f**, **g**), including mean ± SEM. *n* = number of cells, *N* = number of gels. All statistical significance determined by Mann Whitney tests, or by Kruskal-Wallis test (**a**).

We explored reversibility of the *cTnI-G203S* hypermetabolic mitochondrial state arising from cTnI mutation Gly203Ser, by testing the effect of reducing substrate stiffness on *Ψ*_m_ and mitochondrial metabolic activity in cardiac myocytes isolated from *cTnI-G203S* mice with established HCM. *cTnI-G203S* mice exhibit hallmark features of HCM including hypertrophy and hypercontractility from 21 weeks of age^5,6,17,35^. Therefore, 30–50-week-old *cTnI-G203S* cardiac myocytes and age-matched *wt* cardiac myocytes were plated on stiff and soft hydrogels for at least 3 h to mimic innate HCM and healthy conditions respectively. Alterations in cellular stiffness and mitochondrial function were then assessed. Consistent with data collected from 10 to 15-week old *wt* cardiac myocytes, 30–50-week-old *wt* myocytes plated on stiff hydrogels displayed a significant increase in compressive stiffness versus soft hydrogels (*p* < 0.05, Fig. 5a). Additionally, 30–50-week-old *cTnI-G203S* myocytes plated on soft hydrogels displayed a significant decrease in compressive stiffness versus those plated on stiff hydrogels (*p* < 0.05, Fig. 5a). These data demonstrate that the compressive stiffness of *wt* cardiac myocytes may be manipulated to mimic that of *cTnI-G203S* myocytes, and vice versa. Consistent with the HCM phenotype, exaggerated responses were observed in *cTnI-G203S* versus *wt* myocytes when plated on soft or stiff conditions (Fig. 5a). Whilst no alteration in lamin A or vinculin expression was observed, plating *cTnI-G203S* myocytes on soft hydrogels resulted in a significant decrease in β_1_-integrin expression (Fig. 5b–g), implicating integrin as a key mechanosensing protein in *cTnI-G203S* myocytes.

**Fig. 6:**
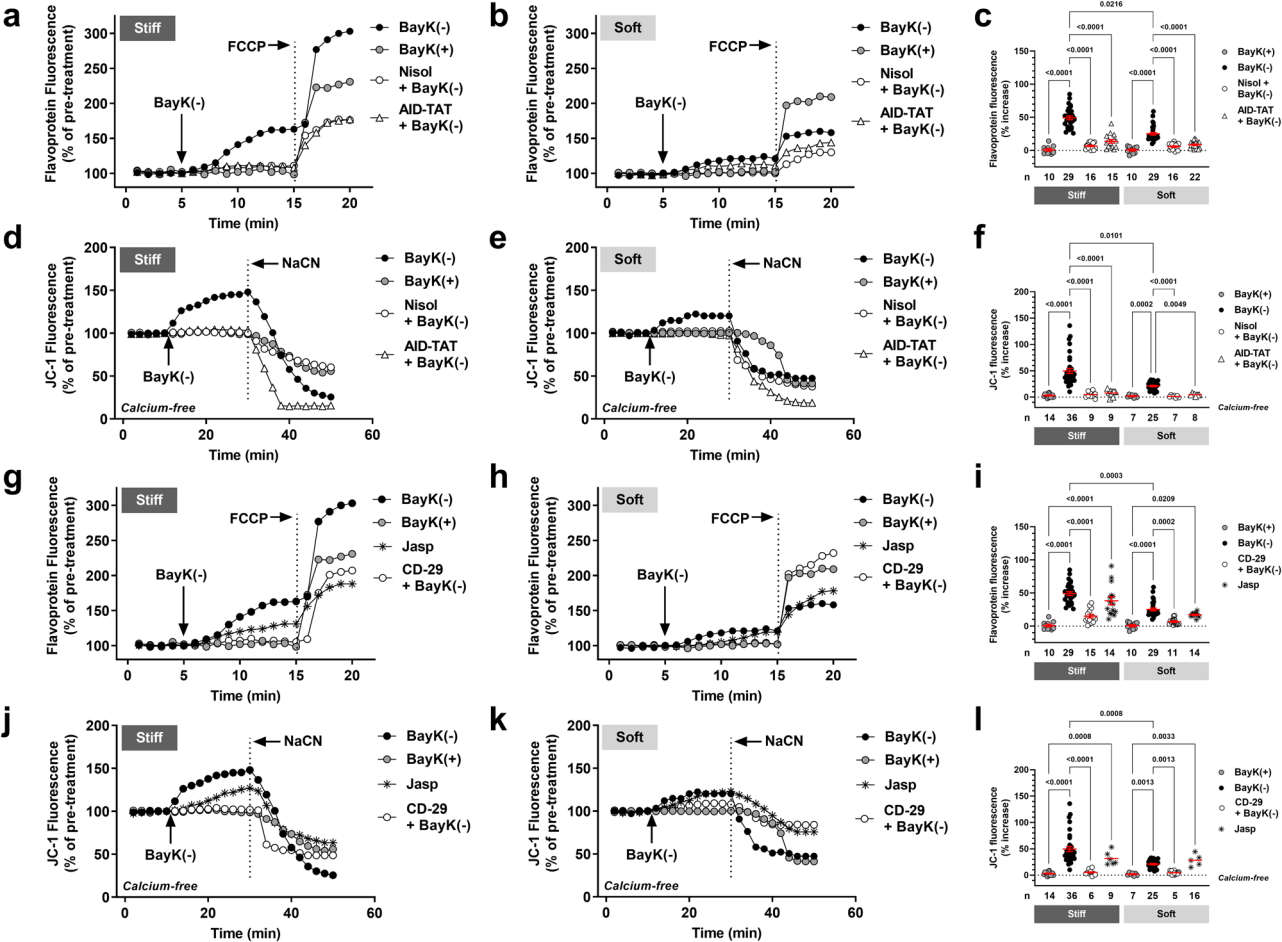
The cardiac L-type calcium channel and integrin participate as partners in mediating the hypermetabolic mitochondrial state exhibited by cTnI-G203S cardiac myocytes. Representative ratiometric flavoprotein (**a**, **b** and **g**, **h**) and JC-1 (**d**, **e** and **j**, **k**) fluorescence recorded cardiac myocytes isolated from cardiomyopathic *cTnI-G203S* mice plated on stiff and soft hydrogels before and after exposure to 10 μM BayK(+), Jasplakinolide (Jasp, 1 μM, 20 min pre-incubation), or 10 μM BayK(−) in the absence or presence of nisoldipine (15 μM, Nisol), AID-TAT (10 μM), or CD-29 (5 μg/ml, 30 min pre-incubation). JC-1 studies were performed under calcium-free conditions (0 mM calcium). Arrows indicate addition of drugs. To confirm signals were mitochondrial in origin, FCCP (50 μM) was applied at the end of each flavoprotein experiment to increase flavoprotein oxidation. NaCN (40 mM) was applied at the end of each JC-1 experiment to collapse *Ψ*_m_. Overall flavoprotein (**c** and **i**) and JC-1 (**f** and **l**) fluorescence for all myocytes (n) exposed to drugs as indicated, including mean ± SEM. All statistical significance determined by Kruskal-Wallis tests.

We also measured alterations in mitochondrial metabolic activity and *Ψ*_m_ in cardiac myocytes isolated from 30–50-week-old *cTnI-G203S* mice plated on stiff and soft hydrogels, in response to activation of the LTCC using BayK(−). We found that *cTnI-G203S* myocytes plated on stiff and soft hydrogels both showed a significant increase in flavoprotein oxidation and *Ψ*_m_ following exposure to BayK(−) versus BayK(+) (Fig. 6a–f). The magnitude of the BayK(−) response was significantly reduced on soft versus stiff hydrogels, indicating that ECM stiffness plays a critical role in regulating alterations in mitochondrial function in *cTnI-G203S* pathophysiology. All BayK(−) induced responses could be attenuated with application of nisoldipine or AID-TAT (Fig. 6a–f), confirming that a structural-functional communication between the LTCC and mitochondria mediates the response. Further to this, BayK(−) induced alterations in flavoprotein oxidation and *Ψ*_m_ were significantly attenuated in in the presence of CD-29, whereas application of jasplakinolide alone was sufficient to induce significant increases in flavoprotein oxidation and *Ψ*_m_ (Fig. 6g–l). Taken together, these data suggest that the cardiac LTCC and integrin may participate as partners in mediating the hypermetabolic mitochondrial state exhibited by *cTnI-G203S* myocytes.

### Alterations in relative cardiac mTOR expression and integrin expression appear to precede cTnI-G203S pathology

**Fig. 7:**
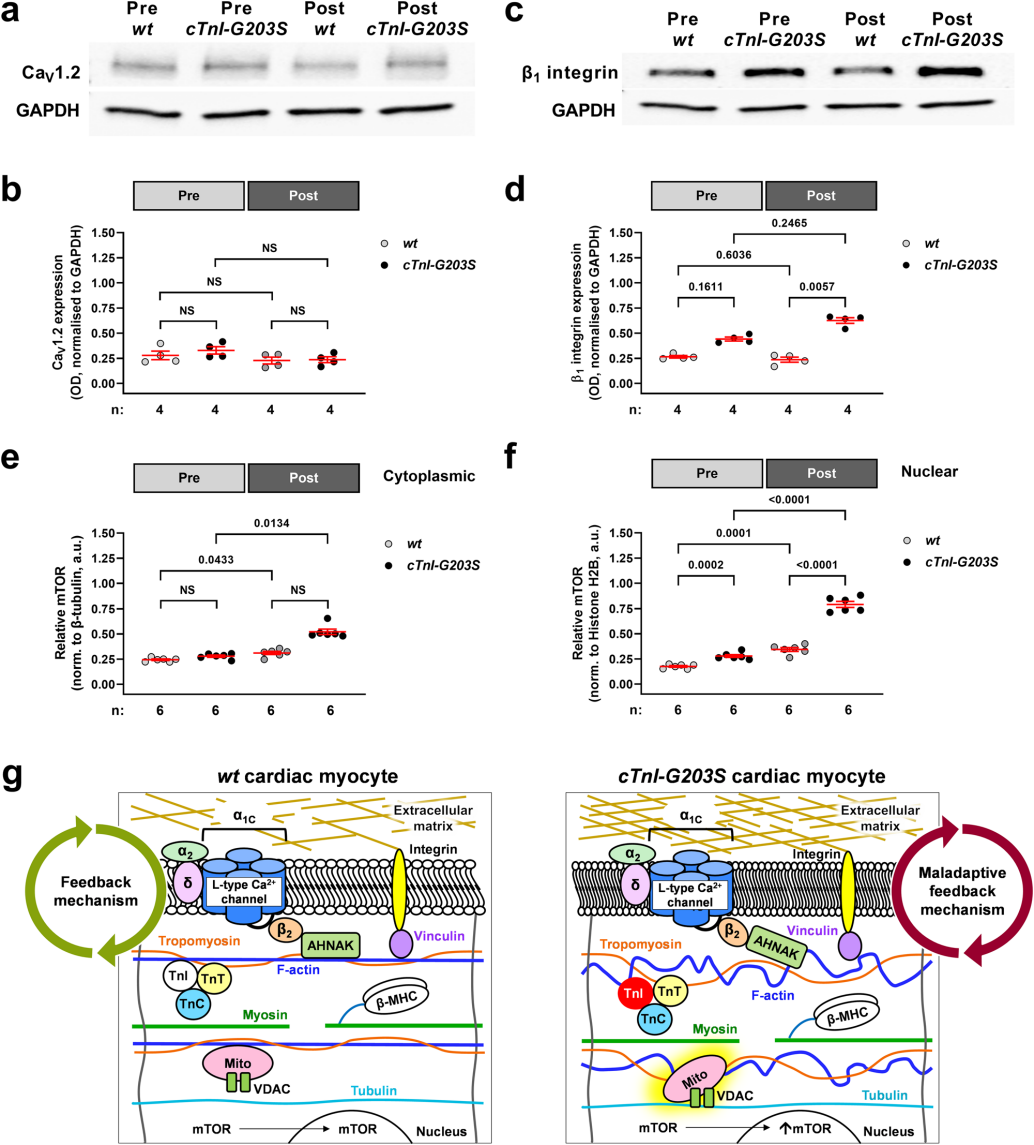
Alterations in relative cardiac mTOR expression and integrin expression appear to precede cTnI-G203S pathology. Immunoblot analysis of L-type calcium channel (**a**, **b**) and β_1_ integrin (**c**, **d**) protein expression performed on total heart homogenate pooled from groups of 5 pre- (10–15-wk-old) or post-cardiomyopathic (30–50-week-old) *cTnI-G203S* mice and age-matched *wt* counterparts. Representative immunoblots probed with L-type calcium channel α_1C_ subunit (Ca_V_1.2, **a**) or β_1_ integrin (**c**) antibody, then GAPDH monoclonal antibody. Densitometry analysis of Ca_V_1.2 (**b**) or β_1_ integrin (**d**) protein expression, normalized to associated GAPDH expression. *n* = number of technical repeats. A Browne-Forsythe and Welch ANOVA (**b**) or Kruskal-Wallis test (**d**) determined statistical significance. Densitometry analysis of relative mTOR expression (calculated as Phospho-mTOR/Total mTOR) performed on cytoplasmic (**e**) and nuclear (**f**) fractions pooled from groups of five pre- or post-cardiomyopathic *cTnI-G203S* mice and age-matched *wt* counterparts. β-tubulin and histone H2B antibodies were used as loading controls for cytoplasmic and nuclear fractions respectively. *n* = number of technical repeats. A Browne-Forsythe and Welch ANOVA (**e**) or Kruskal-Wallis test (**f**) determined statistical significance. **g** Schematic indicating structural-functional link between the L-type calcium channel, cytoskeletal network, mitochondria, integrin and the extracellular matrix in *wt* and *cTnI-G203S* cardiac myocytes. A disrupted cytoskeletal architecture in *cTnI-G203S* cardiac myocytes may trigger a maladaptive feedback mechanism between increased cytoskeletal and extracellular matrix stiffness, resulting in a hypermetabolic mitochondrial state.

To further investigate the role of the LTCC and β_1_ integrin in disease progression, we assessed expression levels of LTCC and β_1_ integrin in whole heart homogenate collected from pre- and post-cardiomyopathic *cTnI-G203S* mice. We found no significant difference in LTCC expression in pre- and post-cardiomyopathic *cTnI-G203S* hearts or age-matched *wt* hearts (Fig. 7a, b and Supplementary Fig. 2). However, alterations in β_1_ integrin expression were observed in pre- and post-cardiomyopathic *cTnI-G203S* hearts versus *wt*, with the most substantial increases occurring in post-cardiomyopathic hearts (Fig. 7c, d and Supplementary Fig. 2). Overall, alterations in integrin expression appear to “worsen” late in *cTnI-G203S* HCM disease progression. This may be indicative of a maladaptive state^36^.

The mammalian target of rapamycin (mTOR) is a calcium-independent signaling pathway, that is a key regulator of protein synthesis, mitochondrial biogenesis and cell metabolism, and has been implicated in the development of HCM^37–39^. Myocytes isolated from *cTnI-G203S* mice exhibit an increase in mitochondrial size and density versus *wt* myocytes, but no alteration in intracellular calcium concentration^2,3^. Therefore here, we tested the involvement of mTOR in the development of *cTnI-G203S* cardiomyopathy. Alterations in LTCC mediated mitochondrial metabolic activity and a resulting hypermetabolic mitochondrial state occurs prior to the development of *cTnI-G203S* pathology^6,17^. To determine whether alterations in protein expression occur early or late in disease progression, we assessed mTOR expression in cytoplasmic and nuclear fractions prepared from hearts of pre- and post-cardiomyopathic *cTnI-G203S* mice. Expression of phosphorylated mTOR (phospho-mTOR) was normalized to total mTOR to yield “relative” mTOR expression (please refer to Supplementary Figs. 3 and 4 for individual phospho-mTOR and total mTOR data). We found that relative mTOR expression was significantly increased in post- versus pre-cardiomyopathic *cTnI-G203S* and *wt* cytoplasmic (Fig. 7e) and nuclear fractions (Fig. 7f); supporting the notion that mTOR may play a role in the ‘normal’ cardiac aging process^37^. However notably, relative mTOR expression was significantly elevated in both pre- and post-cardiomyopathic *cTnI-G203S* nuclear fractions versus age-matched *wt* counterparts (Fig. 7f). These data suggest that activation of mTOR may also contribute to the early phase of the disease. However, further functional studies utilizing the platforms described in this study would be required to confirm a causal role of mTOR in disease progression.

## Discussion

An intracellular “communication breakdown” between the LTCC and mitochondria contributes to a hypermetabolic mitochondrial state arising from cTnI and β-myosin heavy chain gene mutations^6,17,31^. These responses precede development of HCM. Increased ECM protein synthesis has been implicated in the progression of human HCM pathology^20–22^. Here, utilizing an in vitro platform that mimics HCM myocardial stiffness, we demonstrate that alterations in substrate stiffness can modulate the structural-functional communication between the LTCC and mitochondria. This appears to occur via key “linker” sites within the cytoskeletal network, under both healthy and HCM conditions (Figs. 2 and 6). With this, we propose that increased ECM stiffness, upstream of the LTCC, may contribute to the progression of HCM pathophysiology resulting from the Gly203Ser gene mutation. In support of this, engineered heart tissues created by seeding healthy human induced pluripotent stem cell-derived cardiac myocytes onto strips of decellularized myocardium harvested from a swine model of HCM exhibit increased stiffness^21^.

We find that inhibition of mechanosensing protein β_1_-integrin abolished LTCC-induced alterations in mitochondrial metabolic activity, while increasing cytoskeletal stiffness simulated LTCC-induced alterations in mitochondrial function (Figs. 4 and 6). Interestingly, the cardiac LTCC and β_1_-integrin are transmembrane spanning proteins that structurally and functionally associate with F-actin^12,23,25,26^. There is good evidence that cardiac mechanotransduction occurs on both an inside-out and outside-in basis^40,41^. With this, we propose a feedback mechanism between the ECM and cytoskeletal network, via integrin, that assists in regulating alterations in LTCC-mediated mitochondrial function under healthy conditions, yet becomes maladaptive in response to sarcomeric gene mutation Gly203Ser, thereby participating in progression of the HCM disease state (Fig. 7g). To assess the effect of alterations in substrate stiffness on regulation of mitochondrial function by the LTCC under both healthy and HCM conditions, all studies in this paper were performed in quiescent cardiac myocytes. Furthermore, to explore the metabolic regulatory role of the structural-functional link between the LTCC and mitochondria, alterations in Ψ_m_ were performed under calcium-free conditions. Future studies involving measurements of contractility would be required to further elucidate the role of ECM stiffness on the mechanisms identified in this study.

Genetic studies have identified over 1500 different sarcomere gene mutations associated with the development of HCM^4^. A review of studies investigating HCM pathophysiology revealed that while some similarities exist, each mutation appears to lead to mutation-specific pathophysiology^42^. Therefore, further investigation would be required to confirm the involvement of the proposed mechanism in the development of specific HCM causing sarcomeric gene mutations. From a therapeutic perspective, treatment of *cTnI-G203S* mice with AID-TAT restores LTCC kinetics, mitochondrial metabolic activity, and prevents the development of hypertrophy^17^. Other studies involving proteomics analysis of cardiac tissue from HCM patients with identified sarcomeric gene mutations suggest the microtubule network as a potential treatment target^43^. These findings support the notion that targeting downstream cellular responses to ECM stiffness may be an important consideration in the development of preventative therapies^44^. With further characterization, key ‘linker’ sites between the LTCC and mitochondria and/or modifying sarcomeric proteins may represent potential therapeutic targets for the development of HCM treatment approaches for patients with identified sarcomeric gene mutations.

## Methods

### Fabrication and functionalization of hydrogels

#### Fabrication

Polyacrylamide (PA) hydrogels were fabricated based on a previously described method^45^. Pre-polymer solutions for hydrogels mimicking healthy (soft) and HCM (stiff) myocardium were made by combining acrylamide (Bio-Rad), bis-acrylamide (Bio-Rad), Dulbecco’s PBS (Mg^2+^ and Ca^2+^ free) (Life Technologies) and deionized H_2_O (See Table 1 for recipe). Coverslips were cleaned and activated in an ozone cleaner for 1 min on each side (Bioforce Nanosciences UV/Ozone ProCleaner), then functionalized for 5 min in a solution containing 3-(trimethoxysilyl)propyl methacrylate (0.5% V/V) (Merck), glacial acetic acid (3% V/V) and pure ethanol (96.5% V/V). Functionalized coverslips were rinsed in ethanol for 1 min and allowed to dry in air, then coated with dimethydichlorosilane (DCDMS) to create a flat, non-adhesive surface to fabricate hydrogels. A polymer solution was then prepared by adding 10 µl of ammonium persulfate (APS) (Bio-Rad) and 1 µl of N,N,N′,N′- tetramethylethylenediamine (TEMED) (Bio-Rad) to 1 ml of pre-polymer solution. Next, 4 or 12 µl polymer solution was pipetted onto DCDMS coated glass slides to achieve a final height of 50 µm (for 10 mm diameter round or 15 × 15 mm^2^ square coverslips respectively). Functionalized coverslips were then gently placed on top. Hydrogels were allowed 20 min to polymerize before coverslips were removed from DCDMS coated slides and UV sterilized.

**Table 1:** Pre-polymer solution recipes.

	Acrylamide	2% Bis-acrylamide
Soft (Healthy)	10%	0.1%
Stiff (HCM)	8%	0.48%

#### Functionalization

Hydrogels were rinsed three times in 4-(2-hydroxyethyl)-1-piperazineethanesulfonic acid buffer (HEPES) (50 mM, pH 8.5) (Life Technologies), then immersed in N-sulphosuccinimidyl-6-(4′- azido-2′-nitrophenylamino) hexanoate (sulpho-SANPAH; Thermo Scientific) in HEPES (1 mg/ml). Sulfo-SANPAH was activated under UV light (365 nm, 10 min), then removed, and hydrogels rinsed in HEPES. Hydrogels were then immersed in mouse laminin (ThermoFisher 23017015) in HEPES (25 µg/ml) and incubated overnight at 37 ˚C. Prior to use, hydrogels were rinsed with PBS to remove unbound laminin.

### Atomic force microscopy

Atomic force microscopy (AFM) was used to measure the compressive stiffness of hydrogels and single cells, measured in Young’s moduli. Measurements were made with an MFP-3D atomic force microscope (Asylum Research), using 200 µm chromium/gold-coated pyrex-nitride cantilevers with triangular-shaped tips (Nano World model PNP-TR). Measurements were made in 1× PBS (Mg^2+^ and Ca^2+^ free) and probed with 2nN indentations (approach velocity: 2 µm/s, retraction velocity: 10 µm/s). The linear portion of the contact generated force curve was used to determine Young’s modulus through custom-written code in Igor Pro, as previously described^46^. Indentations were made in triplicate to ensure the stability of measurements and averaged to give an average stiffness per indentation. Six points across each hydrogel were chosen at random to be indented. The average of these six indentations was used as an estimate of the overall compressive stiffness of the hydrogel^47^. Two technical repeats per biological repeat were characterized to ensure that hydrogels were of a consistent stiffness. Stiffness of cells was characterized by indenting the center of single cells. Approximately 28 cells were indented per biological repeat^27,48^. For single cell stiffness, four technical repeats were performed per biological repeat. A minimum of four biological repeats were conducted per condition.

### H9C2 cell line

The H9C2 myoblast cell line, isolated from rat ventricular tissue, was supplied by the European Collection of Cell Cultures (ECACC, Salisbury, United Kingdom), and purchased from CellBank Australia (Westmead, NSW, catalog number 88092904). H9C2 cells were cultured as per ECACC instructions. Cells were resuscitated in complete growth media comprised of Dulbecco’s Modified Eagle Medium (DMEM, high glucose, pyruvate, ThermoFisher), supplemented with 4 mM glutamine and 10% fetal calf serum. Cells were seeded at 1–3 × 10,000 cells/cm^2^. Media changes were performed once every 2–3 days. Sub-culturing was performed at ~70–80% confluence. To sub-culture or plate onto PA hydrogels, DMEM was removed and discarded, replaced with 0.25% Trypsin-EDTA (ThermoFisher) and incubated for ~3 min (37 °C) before centrifuging cell suspension at 1200 rpm (5 min). The supernatant was then removed and the pellet resuspended in pre-warmed complete growth media. For PA hydrogels, cells were plated at 10,000 cells per hydrogel (soft or stiff), and incubated for 24–48 h at 37 °C prior to experimentation.

### Adult cardiac myocytes

#### Mouse model

Male mice were used for all studies. Adult cardiac myocytes isolated from mice expressing the human cTnI gene encoding the human HCM causing mutation *cTnI-G203S* were used. The mice develop hallmark features of HCM by 21 weeks^5,6,17,35^. 10–15-week-old (pre-cardiomyopathic) and 30–50-week-old (post-cardiomyopathic) *cTnI-G203S* mice were used for in vitro and ex vivo studies. Age-matched mice expressing the normal human cTnI gene were used as controls (*wt*). Male mice were used to eliminate potential differences in responses due to sex. Experiments were performed in a total of seventy-four 10–15-week-old *wt* mice, ten 10–15-week-old *cTnI-G203S* mice, sixteen 30–50-week-old *wt* mice, and forty-five 30–50-week-old *cTnI-G203S* mice. All animals were randomly assigned to treatment groups. All animal studies were approved by the Animal Ethics Committee of The University of Western Australia in accordance with the *Australian Code of Practice for the Care and Use of Animals for Scientific Purposes* (NHMRC, 8th Edition, 2013).

#### Cell isolation

Adult cardiac myocytes were isolated from *wt* and *cTnI-G203S* mice as previously described^16,17,49^. Mice were anaesthetized with pentobarbitone sodium (240 mg/kg, via intraperitoneal injection) then hearts excised and cannulated onto a Langendorff apparatus via the aorta. Hearts were perfused with Krebs–Henseleit Buffer (KHB) containing (in mM): 120 NaCl, 25 NaHCO_3_, 4.8 KCl, 2.2 MgSO_4_, 1.2 NaH_2_PO_4_ and 11 glucose (pH = 7.35 with O_2_/CO_2_ at 37 °C) for: 4 min at 37 °C, then 3 min in the presence of 2.4 mg/ml collagenase B, then 8 min in the presence of 40 μM calcium. Following perfusion, ventricular tissue was teased apart and triturated to dissociate myocytes into suspension. Myocyte suspension was left to settle for 20 min, supernatant discarded, and myocytes resuspended in calcium free Hepes-Buffered Solution (HBS) containing (in mM): 5.3 KCl, 0.4 MgSO_4_·7H_2_O, 139 NaCl, 5.6 Na_2_HPO_4_·2H_2_O, 5 glucose, 20 Hepes, and 2 glutamine (pH = 7.4 at 37 °C) in the presence or absence of 3 mM EGTA (for 0 mM calcium experiments). For calcium containing experiments, calcium was titrated back to achieve a final extracellular concentration of 1.8 mM. Myocyte suspensions were plated onto hydrogels and incubate for at least 3 h at 37 °C prior to experimentation. All in vitro studies were performed in freshly isolated quiescent myocytes at 37 °C.

### Immunostaining and confocal microscopy

Cells were prepared for confocal imaging based on previously described methods^7,16^. Following plating and incubation, H9C2 cells or adult cardiac myocytes were washed three times with phosphate buffered saline (PBS), containing in mM: 13 KCl, 7.35 KH_2_PO_4_, 0.69 NaCl, 40.4 and 40.4 Na_2_HPO_4_·7H_2_O (pH = 7.4), then fixed with 4% paraformaldehyde (PFA, in PBS, for 15 min), and permeabilized with 0.3% Triton X-100 (15 min). Following permeabilization, cells were incubated in a blocking solution containing 5% goat serum and 5% horse serum (in PBS) for 1 h at 37 °C. Cells were then incubated with primary antibodies lamin A (1:400, Abcam, ab26300) and vinculin (1:200, Abcam, ab130007) in 5% BSA (in PBS, 1 h at 37 °C). Cells were then incubated with rhodamine phalloidin (TRITC, 1:400, ThermoFisher, R415), Goat F(ab) Anti-Rabbit IgG H&L (FITC, 1:200, Abcam ab7050) and Goat Anti-Mouse IgG H&L (Alexa Fluor® 647, 1:200, Abcam ab150115) in 5% BSA (in PBS, 1 h at 37 °C). Alternatively, cells were incubated with a β_1_ integrin primary antibody (1:100, ThermoFisher MA1-06906) in 5% BSA (in PBS, 1 h at 37 °C), followed by Goat anti-Mouse IgG H&L (Alexa Fluor® Plus 488, 1:400, ThermoFisher A32723) in 5% BSA (in PBS, 1 h at 37 °C). Finally, cells were incubated with nucleic acid stain DAPI (1:200 in PBS, Sigma-Aldrich D9542) for 10 min at room temperature before being mounted onto glass slides for imaging. Cells were imaged on an Olympus IX71 inverted fluorescent microscope at 60× magnification.

### Fluorescent detection of in vitro mitochondrial membrane potential (*Ψ*_m_) and mitochondrial flavoprotein oxidation

#### Agonists and antagonists

5,5′,6,6′-tetrachloro-1,1′,3,3′-tetraethylbenzimidazolylcarbocyanine iodide (JC-1) was purchased from Molecular Probes (Eugene, Oregon, USA). Carbonyl cyanide-4-(trifluoromethoxy)phenylhydrazone (FCCP), colchicine, latrunculin A, nisoldipine, (S)-(−)-Bay K8644 (BayK(−)) and sodium cyanide (NaCN) were bought from Merck (Kenilworth, New Jersey, USA). Jasplakinolide was purchased from Cayman Chemical (Ann Arbor, Michigan, USA); (R)-(+)-Bay K8644 (BayK(+)) from BioVision (Milpitas, California, USA); and CD-29 from BD Biosciences (Franklin Lakes, New Jersey, USA). AID-TAT peptide was synthesized using the amino acid sequence QQLEEDLKGYLDWITQAE including a cell-penetrating TAT sequence (RKKRRQRRR) tethered via 6-aminohexanoic acid (AusPep, Tullamarine, Victoria, AUS)^50^. The AHNAK-P4N-TAT peptide was synthesized using the amino acid sequence KGKHGKLKFGTFGGLGSKSKGHYEVT tethered to the TAT sequence as previously described (Mimotopes Pty Ltd, Victoria, AUS).

Mitochondrial membrane potential (*Ψ*_m_) was assessed using fluorescent indicator JC-1 (200 nM, incubation period = 3 h, ex = 480 nm, em = 580/535 nm, interval = 2 min, exposure = 50 ms)^16,17^. At the end of each experiment, mitochondrial electron transport blocker sodium cyanide was applied to collapse *Ψ*_m_, confirming that the JC-1 signal was indicative of *Ψ*_m_ (NaCN, 40 mM). Mitochondrial flavoprotein oxidation was assessed by recording myocyte autofluorescence as previously described (ex = 480 nm, em = 535 nm, interval = 1 min, exposure = 250 ms)^16,17^. Mitochondrial electron transport chain uncoupler FCCP (50 μM) was added at the end of each experiment to increase flavoprotein oxidation, confirming signal was mitochondrial in origin. All in vitro fluorescence was measured on an Andor Zyla SCMOS 5.5 MP camera attached to an inverted Nikon TE2000-U microscope. Ratiometric JC-1 or flavoprotein fluorescent images were quantified using Metamorph 7.10. Regions containing myocytes were manually traced to obtain a fluorescent signal for each cell. An equivalent region not containing cells was used as background and subtracted for each cell. Responses to treatments were reported as a percentage increase or decrease from the pre-treatment (basal) average.

### Immunoblot studies

The protein concentration of all samples was quantified using the Bradford protein assay using BSA as a standard. 25 µg samples were loaded into precast 10% BIO-RAD Mini-PROTEAN® TGX Stain-Free^TM^ SDS-polyacrylamide gel (Bio-RAD Laboratories), then electrophoretically transferred to 0.2 µm nitrocellulose membrane (Trans-Blot® TurboTM Transfer Pack, Bio-RAD Laboratories) using the Bio-RAD Trans-Blot® TurboTM Transfer System. Blots were stripped between each re-probing step using stripping buffer, consisting of (in mM): Tris HCl pH 6.8: 62.5, 2-mercaptoethanol: 100, 2% SDS, 30 min at 50 °C). Quantitative densitometry was performed on images captured using a Chemidoc imaging system (Bio-rad), and ImageJ software. Data are presented as optical density of protein expression, normalized to loading control detected on the same blot.

#### LTCC (Ca_V_1.2) and β_1_ integrin protein

Immunoblot analysis was performed on total heart homogenate pooled from groups of five 10–15-week-old and 30–50-week-old *wt* and *cTnI-G203S* mice. Briefly, snap frozen hearts were weighed, ground into a fine powder in liquid N_2_ using a mortar and pestle, and homogenized in 1:4 RIPA buffer consisting of (in mM): NaCl 150, Tris 50, Na_4_P_2_O_7_ 20, Na_3_VO_4_ 2, NaF 1, 0.5% Na deoxycholate, 1 % Triton X-100, 0.1 % SDS, pH 7.4, supplemented with cOmplete^™^, Mini, EDTA-free Protease (Roche, 4693159001), and PhosStop^TM^ phosphatase (Roche, 4906837001, 1:100 dilution) inhibitor tablets. Total heart homogenate was then centrifuged at 10,000 *g* for 5 min at 4 °C. Blots were probed with the following primary antibodies: rabbit polyclonal anti-Ca_V_1.2 (Alomone, ACC-003, 1:200) or rabbit monoclonal anti-β_1_ integrin (D6S1W) (Cell Signaling Technology 34971, 1:1000). Rabbit monoclonal anti-GAPDH (Cell Signaling Technology, 2118, 1:1000) was used as a loading control. Blots were then probed with polyclonal goat anti-rabbit IgG H&L (HRP) pre-absorbed secondary antibody (Abcam, ab97080, 1:10,000).

#### mTOR

Subcellular fractions were prepared from groups of five 10–15-week-old and 30-50-week-old *wt* and *cTnI-G203S* hearts, an NE-PER^TM^ Nuclear and Cytoplasmic Extraction Kit (ThermoFisher Scientific, 78833), supplemented with cOmplete^™^, Mini, EDTA-free Protease (Roche, 4693159001) and PhosStop^TM^ phosphatase (Roche, 4906837001) inhibitor tablets, according to manufacturer protocols. Blots were probed with the following primary antibodies: Total mTOR, mTOR (7C10) rabbit mAb (Cell Signaling Technology, 2983, 1:1000); Active mTOR, phospho-mTOR (Ser2448) (D9C2) XP® Rabbit mAb (Cell Signaling Technology, 5536, 1:1000); Total SRP6, S6 ribosomal protein (5G10) rabbit mAb (Cell Signaling Technology, 2217, 1:1000); Active SRP6, phospho-S6 ribosomal protein (Ser235/236) (2F9) rabbit mAb (Cell Signaling Technology, 4856, 1:1000). The following primary antibodies were used for loading controls: β-tubulin antibody (Cell Signaling Technology, 2146, 1:500); histone H2B (D2H6) rabbit mAb (Cell Signaling Technology, 12364, 1:1000). Blots were probed with goat anti-rabbit IgG H&L (HRP) preadsorbed secondary antibody (Abcam, ab97080, 1:10000). The same antibodies were used to confirm purity of cytoplasmic and nuclear fractions (Supplementary Fig. 5).

### Antibody validation

#### Rabbit polyclonal anti-Ca_V_1.2 (Alomone, ACC-003)

Species: H, M, R; Applications: WB, ICC, IF, IFC, IHC, IP; Validated by Alomone. Citation: 124 (https://www.alomone.com/p/anti-cav1-2-antibody/ACC-003). *Rabbit monoclonal anti-β*_*1*_ *integrin* (D6S1W, Cell Signaling Technology, 34971): Species: H, M, R; Applications: WB, IHC; Validated by Cell Signaling Technology. Citation: 31 (https://www.cellsignal.com/products/primary-antibodies/integrin-b1-d6s1w-rabbit-mab/34971). *Rabbit monoclonal anti-GAPDH* (Cell Signaling Technology, 2118): Species: H, M, R, Mk, B, Pg; Applications: WB, IHC, IF, F; Validated by Cell Signaling Technology. Citation: 5360 (https://www.cellsignal.com/products/primary-antibodies/gapdh-14c10-rabbit-mab/2118). *Total mTOR* (mTOR) (7C10, rabbit mAb, Cell Signaling Technology, 2983): Species: H, M, R, Mk; Applications: WB, IP, IHC, IF, F; Validated by Cell Signaling Technology. Citation: 1837 (https://www.cellsignal.com/products/primary-antibodies/mtor-7c10-rabbit-mab/2983). *Active mTOR* (phospho-mTOR) (Ser2448, D9C2, XP® Rabbit mAb, Cell Signaling Technology, 5536): Species: H, M, R, Mk; Applications: WB, IP, IF; Validated by Cell Signaling Technology. Citation: 1356 (https://www.cellsignal.com/products/primary-antibodies/phospho-mtor-ser2448-d9c2-xp-rabbit-mab/5536). *Total SRP6* (S6 ribosomal protein, 5G10, rabbit mAb, Cell Signaling Technology, 2217): Species: H, M, R, Mk; Applications: WB, IHC, IF; Validated by Cell Signaling Technology. Citation: 1610 (https://www.cellsignal.com/products/primary-antibodies/s6-ribosomal-protein-5g10-rabbit-mab/2217). *Active SRP6* (phospho-S6 ribosomal protein, Ser235/236, 2F9, rabbit mAb, Cell Signaling Technology, 4856): Species: H, M, R, Mk; Applications: WB, IF, F; Validated by Cell Signaling Technology. Citation: 255 (https://www.cellsignal.com/products/primary-antibodies/phospho-s6-ribosomal-protein-ser235-236-2f9-rabbit-mab/4856). β-tubulin antibody (Cell Signaling Technology, 2146): Species: H, M, R, Mk, Z, B; Applications: WB, IHC, IF, F; Validated by Cell Signaling Technology. Citation: 676 (https://www.cellsignal.com/products/primary-antibodies/b-tubulin-antibody/2146). Histone H2B (D2H6, rabbit mAb, Cell Signaling Technology, 12364): Species: H, M, R, Mk; Applications: WB, IHC, ChiP; Validated by Cell Signaling Technology. Citation: 33 (https://www.cellsignal.com/products/primary-antibodies/histone-h2b-d2h6-rabbit-mab/12364).

### Statistics and reproducibility

Results are reported as mean ± SEM. For nonparametric data, statistical significance was accepted at *P* < 0.05 using the Mann Whitney test (two-sided) or Kruskal-Wallis test (one-way) for multiple comparisons. For parametric data, statistical significance was accepted at *P* < 0.05 using a Brown-Forsythe and Welch ANOVA test (one-way) (GraphPad Prism version 9.1.2). Number of replicates and statistical comparisons are specified in figures.

Power and Sample Size software (PS) was used to determine sample size taking into consideration inter-animal variability and intra-animal variability. With this, it was determined that responses would need to be studied in 10 mice from each mouse strain/genotype in order to reject the null hypothesis that the population means of experimental groups are equal with probability/power of 0.95 (type I error probability = 0.05). Of note, *p* values resulting from all in vitro data ranged from *p* < 0.0001 through to *p* < 0.0216, indicating that the sample sizes utilized were sufficient to exceed the set significance level.

No data obtained from live cells were excluded. All attempts at replication in live cells were successful. For in vitro studies, experiments involving the use of different agonists/antagonists were performed across different experimental days, in a random manner. Additionally, two individuals ran experiments concurrently, on two different fluorescent experimental set ups. One individual was privy to the agonists/antagonists being applied to ensure correct agents were applied in the correct order. The other individual was blinded. Data were reliably replicated between individuals in all instances.

### Reporting summary

Further information on research design is available in the Nature Portfolio Reporting Summary linked to this article.

## Supplementary information

Supplementary Information

Description of Additional Supplementary Files

Supplementary Data

Reporting Summary

## Data Availability

All data generated or analyzed during this study are included in this published article (and the Supplementary Information file). Source data can be obtained in the Supplementary Data file.
