# Supplementary material for: A maladaptive feedback mechanism between the extracellular matrix and cytoskeleton contributes to hypertrophic cardiomyopathy pathophysiology
Source: Commun Biol. 2023 Jan 3;6:4. doi: 10.1038/s42003-022-04278-9 (PMC9810744; doi:10.1038/s42003-022-04278-9)
Supplement: Supplementary file 1 — Supplementary Information [file 42003_2022_4278_MOESM1_ESM.docx]

Supplementary Information

**A maladaptive feedback mechanism between the extracellular matrix and cytoskeleton contributes to hypertrophic cardiomyopathy pathophysiology**

Helena M. Viola,^1^* Caitlyn Richworth,^1^ Tanya Solomon,^1^ Ian L. Chin,^1^ Henrietta Cserne Szappanos,^1^ Srinivasan Sundararaj,^3^ Dmitry Shishmarev,^3^ Marco G. Casarotto,^3^ Yu Suk Choi,^1^ Livia C. Hool^1,2^*

*Corresponding author. Email: helena.viola@uwa.edu.au and livia.hool@uwa.edu.au

**This file includes:**

Supplementary Figures 1 to 5

**
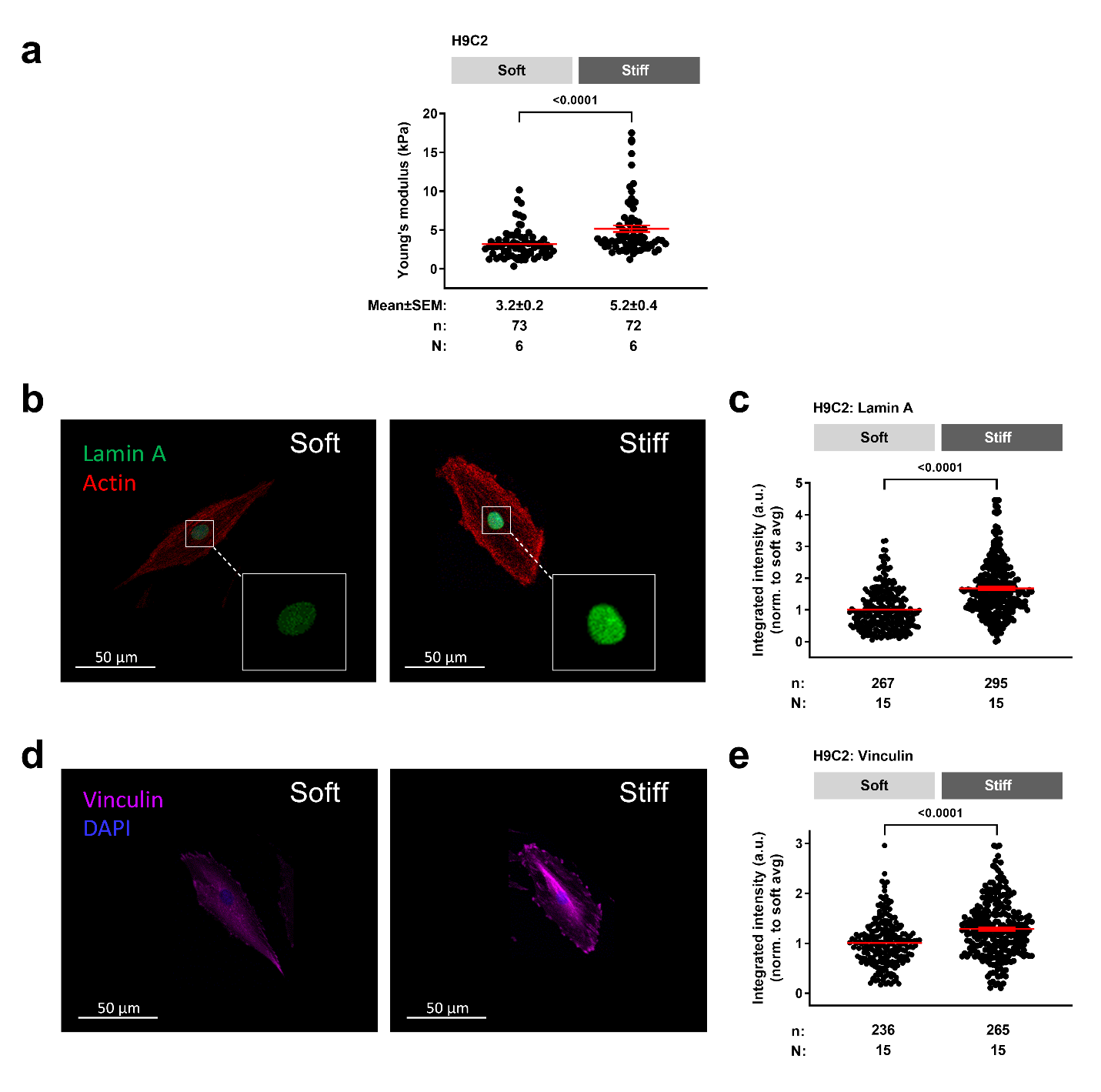
**

**Supplementary Figure 1:** **A polyacrylamide hydrogel platform to regulate cell stiffness.** (**a**) Compressive stiffness (in kPa) of H9C2 cells under manufactured conditions (soft and stiff), including mean ± SEM. (**b-e**) Representative images and quantitated data points for H9C2 cells plated on soft and stiff hydrogels stained for lamin A (**b-c**) and vinculin (**d-e**), including mean ± SEM. For gel work, n = number of indentations, N = number of gels. For cell work, n = number of cells, N = number of gels. Statistical significance determined by Mann Whitney tests.


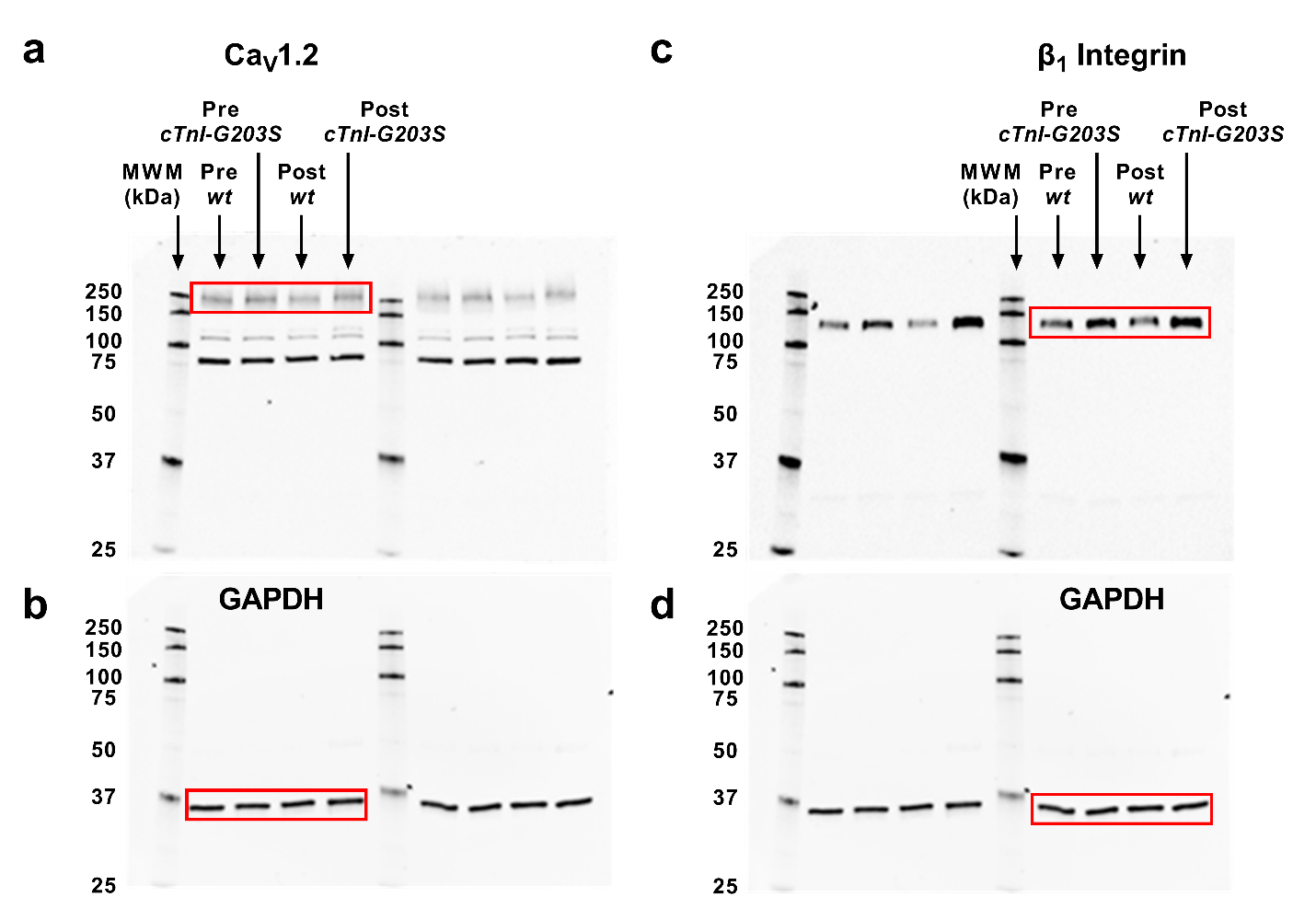


**Supplementary Figure 2: Complete immunoblots for analysis of L-type calcium channel and β_1_ integrin protein expression.** **a-b**, Representative immunoblots probed with L-type calcium channel α_1C_ subunit (Ca_V_1.2, **a**) then GAPDH monoclonal antibody (**b**). **c-d**, Representative immunoblots probed with β_1_ integrin (**c**), then GAPDH monoclonal (**d**) antibody. Red boxes indicate bands of interest.

**
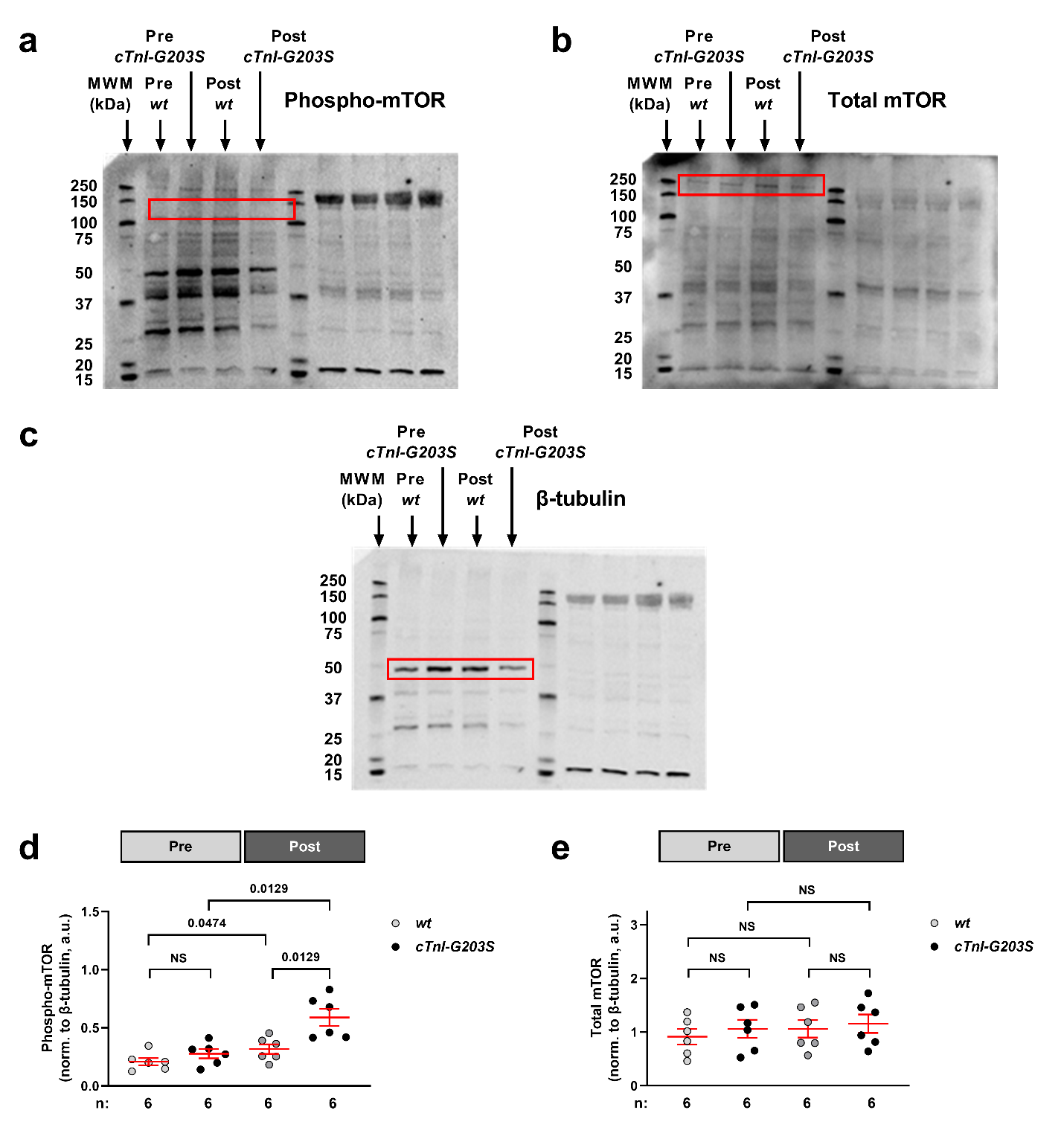
**

**Supplementary Figure 3: Phospo-mTOR and total mTOR expression in cytoplasmic fractions produced from *wt* and *cTnI-G203S* hearts. a-c**, Representative immunoblots performed on cytoplasmic fractions pooled from groups of 5 10-15-week-old (pre) or 30-50-week-old (post) *wt* and *cTnI-G203S* mice probed with phospho-mTOR (**a**), total mTOR (**b**), then β-tubulin (**c**) antibody. **d-e**, Densitometry analysis of phospho-mTOR (**d**) and total mTOR (**e**) protein expression, normalized to associated β-tubulin expression. n = number of technical repeats. All statistical significance determined by Browne-Forsythe and Welch ANOVA.


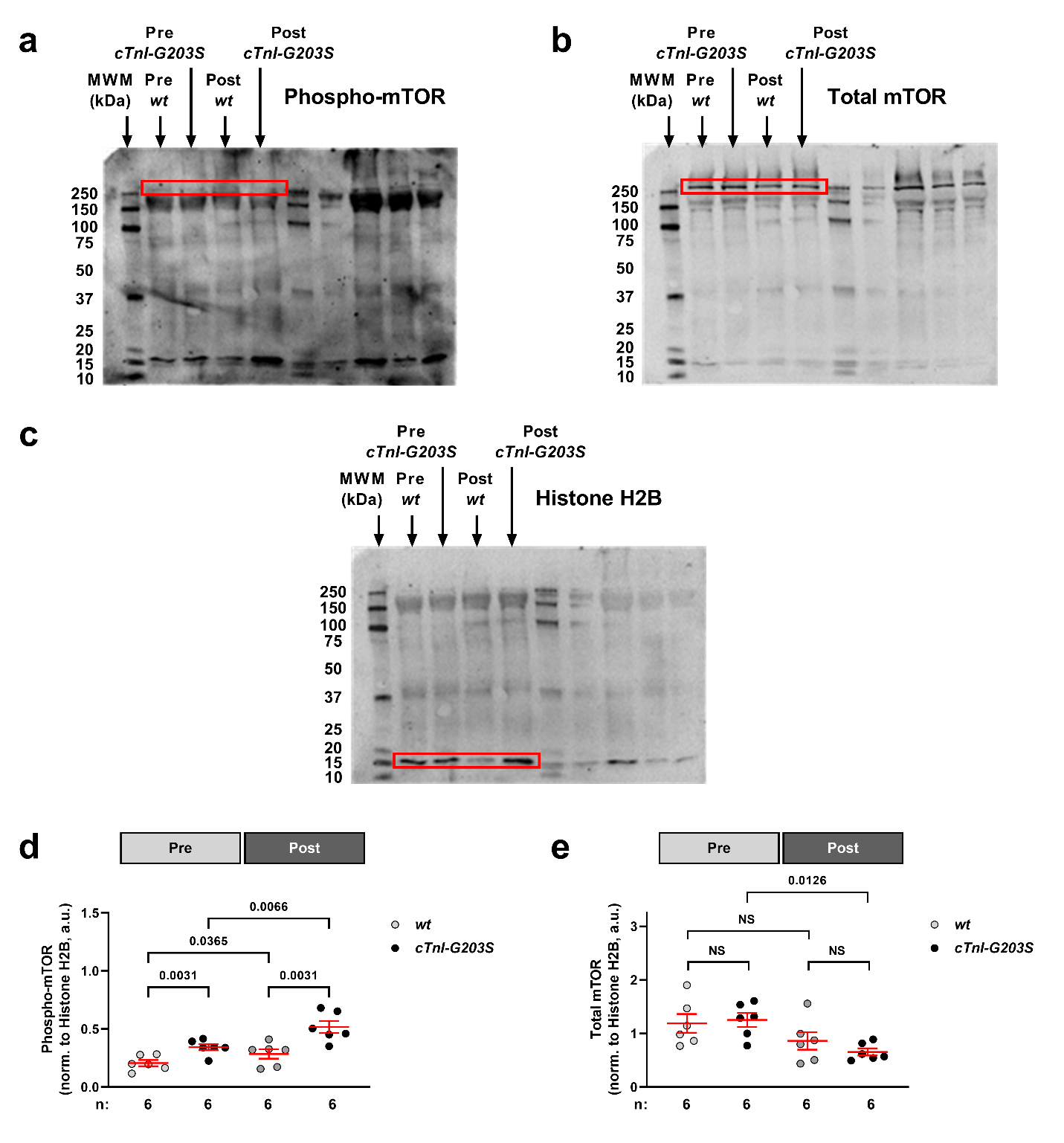


**Supplementary Figure 4: Phospo-mTOR and total mTOR expression in nuclear fractions produced from *wt* and *cTnI-G203S* hearts. a-c**, Representative immunoblots performed on nuclear fractions pooled from groups of 5 10-15-week-old (pre) or 30-50-week-old (post) *wt* and *cTnI-G203S* mice probed with phospho-mTOR (**a**), total mTOR (**b**), then histone H2B (**c**) antibody. **d-e**, Densitometry analysis of phospho-mTOR (**d**) and total mTOR (**e**) protein expression, normalized to associated histone H2B expression. n = number of technical repeats. All statistical significance determined by Browne-Forsythe and Welch ANOVA.


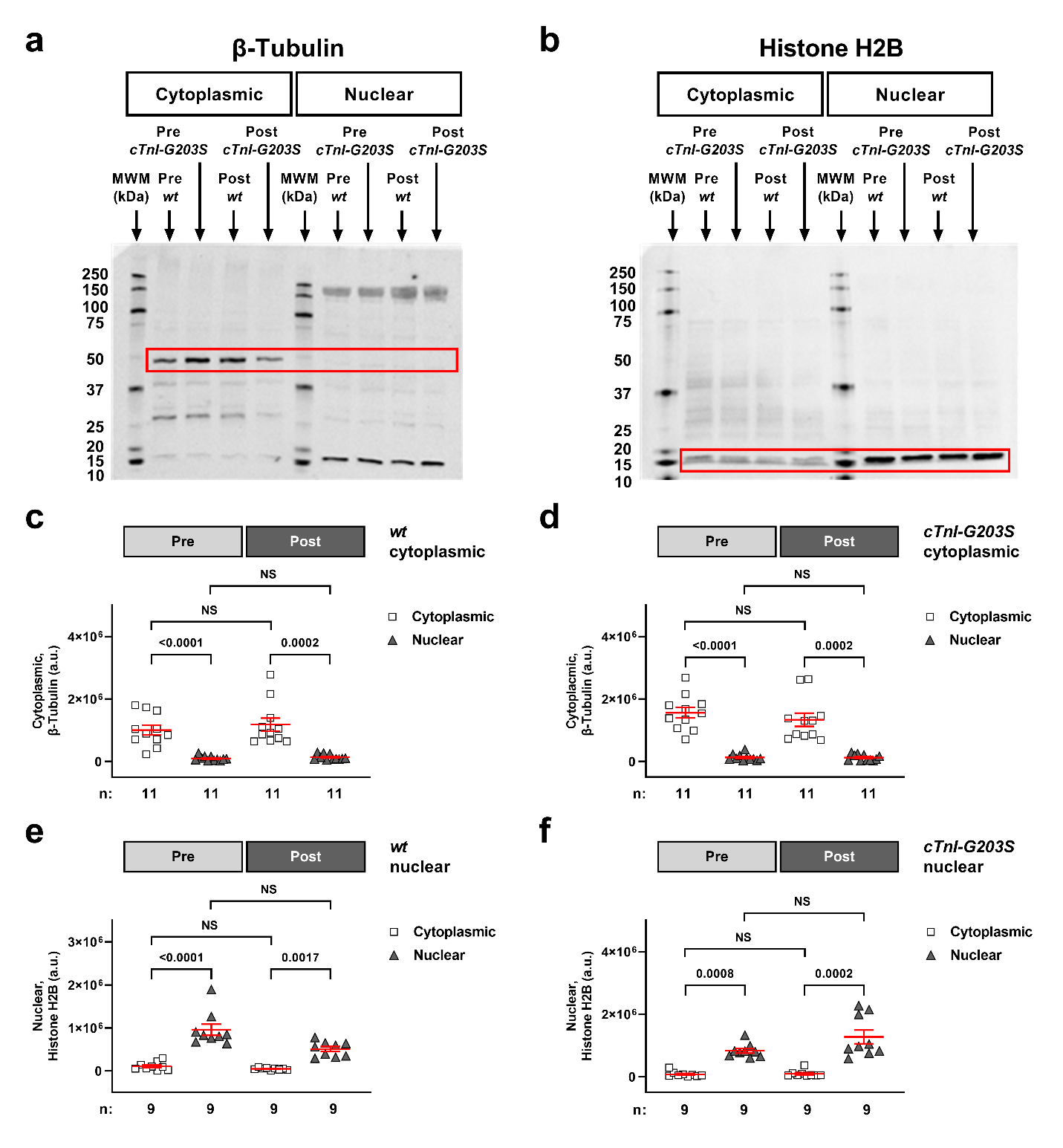


**Supplementary Figure 5: Purity test of cytoplasmic and nuclear fractions prepared from *wt* and *cTnI-G203S* hearts.** **a-b,** Representative immunoblots of cytoplasmic and nuclear fractions pooled from groups of 5 10-15-week-old (pre) or 30-50-week-old (post) *wt* or *cTnI-G203S* mice probed with β-tubulin (**a**, cytoplasmic marker) and histone H2B (**b**, nuclear marker) antibodies as indicated. **c-d**, Densitometry analysis of β-tubulin protein expression in *wt* (**c**) or *cTnI-G203S* (**b**) samples. **e-f,** Densitometry analysis of histone H2B protein expression in *wt* (**e**) or *cTnI-G203S* (**f**) samples. n = number of technical repeats. All statistical significance determined by Kruskal-Wallis tests.
