# Supplementary material for: A maladaptive feedback mechanism between the extracellular matrix and cytoskeleton contributes to hypertrophic cardiomyopathy pathophysiology
Source: Commun Biol. 2023 Jan 3;6:4. doi: 10.1038/s42003-022-04278-9 (PMC9810744; doi:10.1038/s42003-022-04278-9)
Supplement: Supplementary file 2 — Description of Additional Supplementary Files [file 42003_2022_4278_MOESM2_ESM.pdf]

Description of Additional Supplementary Files

File name: Supplementary Data

Description: Source data behind the graphs in the paper.
