# Supplementary material for: A maladaptive feedback mechanism between the extracellular matrix and cytoskeleton contributes to hypertrophic cardiomyopathy pathophysiology
Source: Commun Biol. 2023 Jan 3;6:4. doi: 10.1038/s42003-022-04278-9 (PMC9810744; doi:10.1038/s42003-022-04278-9)
Supplement: Supplementary file 4 — Reporting Summary [file 42003_2022_4278_MOESM4_ESM.pdf]

nature research

Corresponding author(s): Helena M. VIola

Last updated by author(s): Nov 9, 2022

Reporting Summary

Nature Research wishes to improve the reproducibility of the work that we publish. This form provides structure for consistency and transparency in reporting. For further information on Nature Research policies, see our Editorial Policies and the Editorial Policy Checklist.

Statistics

For all statistical analyses, confirm that the following items are present in the figure legend, table legend, main text, or Methods section.

n/a	Confirmed	
☐	☒	The exact sample size (*n*) for each experimental group/condition, given as a discrete number and unit of measurement
☐	☒	A statement on whether measurements were taken from distinct samples or whether the same sample was measured repeatedly
☐	☒	The statistical test(s) used AND whether they are one- or two-sided<br>*Only common tests should be described solely by name; describe more complex techniques in the Methods section.*
☒	☐	A description of all covariates tested
☐	☒	A description of any assumptions or corrections, such as tests of normality and adjustment for multiple comparisons
☐	☒	A full description of the statistical parameters including central tendency (e.g. means) or other basic estimates (e.g. regression coefficient) AND variation (e.g. standard deviation) or associated estimates of uncertainty (e.g. confidence intervals)
☐	☒	For null hypothesis testing, the test statistic (e.g. *F*, *t*, *r*) with confidence intervals, effect sizes, degrees of freedom and *P* value noted<br>*Give P values as exact values whenever suitable.*
☒	☐	For Bayesian analysis, information on the choice of priors and Markov chain Monte Carlo settings
☒	☐	For hierarchical and complex designs, identification of the appropriate level for tests and full reporting of outcomes
☒	☐	Estimates of effect sizes (e.g. Cohen's *d*, Pearson's *r*), indicating how they were calculated

Our web collection on statistics for biologists contains articles on many of the points above.

Software and code

Policy information about availability of computer code

Data collection	N/A
Data analysis	N/A

For manuscripts utilizing custom algorithms or software that are central to the research but not yet described in published literature, software must be made available to editors and reviewers. We strongly encourage code deposition in a community repository (e.g. GitHub). See the Nature Research guidelines for submitting code & software for further information.

Data

Policy information about availability of data

All manuscripts must include a data availability statement. This statement should provide the following information, where applicable:

- Accession codes, unique identifiers, or web links for publicly available datasets
- A list of figures that have associated raw data
- A description of any restrictions on data availability

All data needed to evaluate the conclusions in the paper are present in the paper and/or the Supplementary Materials

Field-specific reporting

Please select the one below that is the best fit for your research. If you are not sure, read the appropriate sections before making your selection.

☒ Life sciences ☐ Behavioural & social sciences ☐ Ecological, evolutionary & environmental sciences

For a reference copy of the document with all sections, see nature.com/documents/nr-reporting-summary-flat.pdf

Life sciences study design

All studies must disclose on these points even when the disclosure is negative.

Sample size	Power and Sample Size software (PS) has been used to determine sample size taking into consideration inter-animal variability and intra-animal variability. With this, it has been determined that responses would need to be studied in 10 mice from each mouse strain / genotype in order to reject the null hypothesis that the population means of experimental groups are equal with probability/power of 0.95 (type I error probability=0.05). It should be noted that here, p values resulting from in vitro data ranged from p<0.0001 through to p<0.0216. Therefore, the sample sizes utilized were sufficient to exceed the set significance level (5%), falling at or below 2% significance.
Data exclusions	No data obtained from live cells were excluded.
Replication	All attempts at replication in live cells were successful.
Randomization	For in vitro studies, experiments involving the use of different agonists/antagonists were performed across different experimental days, in a random manner (see blinding statement below).
Blinding	For in vitro studies, two individuals ran experiments concurrently, on two different fluorescent experimental set ups. One individual was privy to the agonists/antagonists being applied to ensure correct agents were applied in the correct order. The other individual was blinded. Data was reliably replicated between individuals in all instances.

Reporting for specific materials, systems and methods

We require information from authors about some types of materials, experimental systems and methods used in many studies. Here, indicate whether each material, system or method listed is relevant to your study. If you are not sure if a list item applies to your research, read the appropriate section before selecting a response.

Materials & experimental systems

n/a	Involved in the study	
☐	☒	Antibodies
☒	☐	Eukaryotic cell lines
☒	☐	Palaeontology and archaeology
☐	☒	Animals and other organisms
☒	☐	Human research participants
☒	☐	Clinical data
☒	☐	Dual use research of concern

Methods

n/a	Involved in the study	
☒	☐	ChIP-seq
☒	☐	Flow cytometry
☒	☐	MRI-based neuroimaging

Antibodies

Antibodies used	PRIMARY ANTIBODIES: LTCC, Rabbit polyclonal anti-CaV1.2 (Alomone, ACC-003, Lot: ACC003AN6702); Integrin, Rabbit monoclonal anti-β1 integrin (Cell Signaling Technology, 34971, Clone: D6S1W, Lot: 3); Total mTOR, mTOR (7C10) rabbit mAb (Cell Signaling Technology, 2983, Clone: 7C10, Lot: 19); Active mTOR, phospho-mTOR (Ser2448) (D9C2) XP® Rabbit mAb (Cell Signaling Technology, 5536, Clone: D9C2, Lot: 9); Total SRP6, S6 ribosomal protein (5G10) rabbit mAb (Cell Signaling Technology, 2217, Clone: 5G10, Lot; 10); Active SRP6, phospho-S6 ribosomal protein (Ser235/236) (2F9) rabbit mAb (Cell Signaling Technology, 4856, Clone: 2F9, Lot: 9). LOADING CONTROLS: Rabbit monoclonal anti-GAPDH (Cell Signaling Technology, 2118, Clone: D4C6R Lot: 10); β-tubulin antibody (Cell Signaling Technology, 2146, Lot: 9); Histone H2B (D2H6) rabbit mAb (Cell Signaling Technology, 12364, Clone: D2H6, Lot: 3). Polyclonal goat anti-rabbit IgG H&L (HRP) pre-absorbed (Abcam, ab97080).
Validation	● Rabbit polyclonal anti-CaV1.2 (Alomone, ACC-003) (Species: H, M, R; Applications: WB, ICC, IF, IFC, IHC, IP), KO validated by Alomone. Citation: 124, see company website https://www.alomone.com/p/anti-cav1-2-antibody/ACC-003<br>● Rabbit monoclonal anti-β1 integrin (D6S1W) (Cell Signaling Technology, 34971) (Species: H M R, Applications: WB, IHC) Validated by Cell Signaling Technology. Citation: 31, see company website https://www.cellsignal.com/products/primary-antibodies/integrin-b1-d6s1w-rabbit-mab/34971<br>● Rabbit monoclonal anti-GAPDH (Cell Signaling Technology, 2118) (Species: H M R Mk B Pg, Applications: WB, IHC, IF, F) Validated by Cell Signaling Technology. Citation: 5360, see company website https://www.cellsignal.com/products/primary-antibodies/gapdh-14c10-rabbit-mab/2118<br>● Total mTOR: mTOR (7C10) rabbit mAb (Cell Signaling Technology, 2983) (Species: H M R Mk, Applications: WB, IP, IHC, IF, F)

Validated by Cell Signaling Technology. Citation: 1837, see company website https://www.cellsignal.com/products/primary-antibodies/mtor-7c10-rabbit-mab/2983
● Active mTOR: phospho-mTOR (Ser2448) (D9C2) XP® Rabbit mAb (Cell Signaling Technology, 5536) (Species: H M R Mk, Applications: WB, IP, IF) Validated by Cell Signaling Technology. Citation: 1356, see company website https://www.cellsignal.com/products/primary-antibodies/phospho-mtor-ser2448-d9c2-xp-rabbit-mab/5536
● Total SRP6: S6 ribosomal protein (5G10) rabbit mAb (Cell Signaling Technology, 2217) (Species: H M R Mk, Applications: WB, IHC, IF) Validated by Cell Signaling Technology. Citation: 1610, see company website https://www.cellsignal.com/products/primary-antibodies/s6-ribosomal-protein-5g10-rabbit-mab/2217
● Active SRP6: phospho-S6 ribosomal protein (Ser235/236) (2F9) rabbit mAb (Cell Signaling Technology, 4856) (Species: H M R Mk, Applications: WB, IF, F) Validated by Cell Signaling Technology. Citation: 255, see company website https://www.cellsignal.com/products/primary-antibodies/phospho-s6-ribosomal-protein-ser235-236-2f9-rabbit-mab/4856
● β-tubulin antibody (Cell Signaling Technology, 2146) (Species: H M R Mk Z B, Applications: WB, IHC, IF, F) Validated by Cell Signaling Technology. Citation: 676, see company website https://www.cellsignal.com/products/primary-antibodies/b-tubulin-antibody/2146
● Histone H2B (D2H6) rabbit mAb (Cell Signaling Technology, 12364) (Species: H M R Mk, Applications: WB, IHC, ChiP) Validated by Cell Signaling Technology. Citation: 33, see company website https://www.cellsignal.com/products/primary-antibodies/histone-h2b-d2h6-rabbit-mab/12364

Animals and other organisms

Policy information about studies involving animals; ARRIVE guidelines recommended for reporting animal research

Laboratory animals	Adult cardiac myocytes isolated from male mice expressing the human cTnI gene encoding the human HCM causing mutation cTnI-G203S were used. Age-matched male mice expressing the normal human cTnI gene were used as controls (wt). 10-15-week-old and 30-50-week-old cTnI-G203S and wt male mice were used for in vitro and ex vivo studies as outlined throughout the manuscript.
Wild animals	N/A
Field-collected samples	N/A
Ethics oversight	All animal studies were approved by the Animal Ethics Committee of The University of Western Australia in accordance with the Australian Code of Practice for the Care and Use of Animals for Scientific Purposes (NHMRC, 8th Edition, 2013).

Note that full information on the approval of the study protocol must also be provided in the manuscript.
